# Artificial symbiont replacement in a vertically transmitted plant symbiosis reveals a role for microbe–microbe interactions in enforcing specificity

**DOI:** 10.1093/ismejo/wraf177

**Published:** 2025-08-19

**Authors:** Léa Ninzatti, Thibault G Sana, Tessa Acar, Sandra Moreau, Marie-Françoise Jardinaud, Guillaume Marti, Olivier Coen, Aurelien L Carlier

**Affiliations:** Laboratoire des Interactions Plantes-Microbes-Environnement (LIPME), Université de Toulouse, INRAE, CNRS, Castanet-Tolosan F-31326, France; Laboratoire des Interactions Plantes-Microbes-Environnement (LIPME), Université de Toulouse, INRAE, CNRS, Castanet-Tolosan F-31326, France; Université de Bordeaux, Pessac F-33600, France; Structural Biology of Biofilms Group, European Institute of Chemistry and Biology (IECB), 2 Rue Robert Escarpit, Pessac F-33600, France; Laboratoire des Interactions Plantes-Microbes-Environnement (LIPME), Université de Toulouse, INRAE, CNRS, Castanet-Tolosan F-31326, France; Laboratory of Microbiology, Ghent University, Ghent B-9000, Belgium; Laboratoire des Interactions Plantes-Microbes-Environnement (LIPME), Université de Toulouse, INRAE, CNRS, Castanet-Tolosan F-31326, France; Laboratoire des Interactions Plantes-Microbes-Environnement (LIPME), Université de Toulouse, INRAE, CNRS, Castanet-Tolosan F-31326, France; Metatoul-AgromiX Platform, LRSV, Université de Toulouse, CNRS, UT3, INP, Toulouse F-31077, France; MetaboHUB-MetaToul, National Infrastructure of Metabolomics and Fluxomics, Toulouse F-31077, France; Laboratoire des Interactions Plantes-Microbes-Environnement (LIPME), Université de Toulouse, INRAE, CNRS, Castanet-Tolosan F-31326, France; Laboratoire des Interactions Plantes-Microbes-Environnement (LIPME), Université de Toulouse, INRAE, CNRS, Castanet-Tolosan F-31326, France; Laboratory of Microbiology, Ghent University, Ghent B-9000, Belgium

**Keywords:** Symbiosis, endophyte, T6SS, hereditary, transmission

## Abstract

Some plants engage in permanent, vertically transmitted symbioses with bacteria. Often, these bacteria are hosted extracellularly within structures on the leaves, where they produce specialized bioactive metabolites that benefit their host. These associations are highly specific, with one plant species associating with a single bacterial species, but little is known about how these symbioses originate and how specificity is maintained. In this study, we show that the symbiotic association between a wild yam and a bacterium can be manipulated experimentally and that bacteria-free plants are open to colonization by environmental bacteria. Through metabolic profiling, we show that the endophytic niche is rich in organic acids and intermediates of the tricarboxylic acid cycle cycle. Environmental bacteria capable of utilizing these acids, such as the soil bacterium *Pseudomonas putida*, readily colonize aposymbiotic plants. However, successful colonization is contingent upon the absence of the vertically transmitted symbiont or the impairment of its type VI secretion system. Unexpectedly for a vertically transmitted symbiosis, these findings suggest that microbe–microbe interactions, including antagonism, may play a crucial role in maintaining the specificity of an association. However, low transmission rates of synthetic symbionts provide evidence that transmission barriers or bottlenecks may still occur, further enforcing partner fidelity. Together, these results highlight the complexity of mechanisms underlying mutualistic associations, and provide insights into the evolution of bacterial leaf symbiosis.

## Introduction

Mutualistic interactions are generally costly to maintain, and a degree of specificity is often important for the maintenance of a beneficial holobiont. Specificity between symbiotic partners may be driven by a combination of three factors: the mode of acquisition of the symbiont, environmental selection (e.g. via restrictive nutrients or physico-chemical properties within the niche), and mechanisms underlying recognition between the host and its symbiont [1]. Partner specificity may be achieved by a combination of an aposymbiotic phase, during which the host exists without the partner, and selective barriers allowing the establishment of a cooperative symbiont. For example, in the well-studied legume-*Rhizobium* model, plants recruit their symbionts from the environment in a multi-step process. In this case, partner selection relies on the exchange of signaling molecules (flavonoids and Nod factors), and the presence of specific structures on the surface of the bacterial cell [2, 3]. Similarly, the bobtail squid *Euprymna scolopes* recruits *Vibrio fischeri* symbionts into ciliated crypts from surrounding sea water. Microbe-associated molecular patterns (MAMPs) derived from the cell surface of *V. fischeri* trigger morphogenesis of the light organ. Importantly, host tissues contain anti-microbials, which create a selective environment favoring *V. fischeri* [4].

Microbe–microbe interactions may also play a key role in the establishment of a symbiosis, where a host may foster competition between a preferred symbiont and nonproductive microorganisms [5]. This phenomenon is known as interference competition, and is a mechanism that underlies partner choice in systems in, which hosts acquire bacteria from the environment at the juvenile stage such as the attine ants/*Streptomyces* and the *Riptortus pedestris/Burkholderia* symbioses [6, 7]. Finally, some symbionts are also transmitted vertically from mother to offspring. Vertical transmission links the evolutionary fates of the host and symbionts, which promotes the evolution of mutualistic interactions through partner-fidelity feedback (i.e. a mechanism that promotes cooperation between species through mutual dependence) [8]. Moreover, vertical transmission usually occurs at a single stage of the lifecycle (e.g. by infecting the germline), thus sheltering symbionts from competition [9]. Strictly vertically transmitted symbionts do not need to be selected from an environmental population, and sophisticated mechanisms to enforce specificity are thought not to be necessary.

Hereditary symbiosis is common in animals, especially insects, but much less described in plants. However, some plants engage in permanent, strictly specific associations with bacteria within their leaves, a phenomenon known as leaf symbiosis. Leaf symbiosis has been described in ~500 plant species, mostly in the Primulaceae and Rubiaceae families [10, 11]. The bacteria, mainly belonging to the family *Burkholderiaceae*, are present in leaf nodules that can take various shapes [12]. Several lines of evidence indicate that the leaf symbionts of some species have a defensive role. For example, *Candidatus* Caballeronia kirkii (Ca. *C. kirkii*); the symbiont of *Psychotria kirkii* (Rubiaceae), is linked to the accumulation of bioactive cyclitols in the plant. Of these, kirkamide displays cytotoxic and insecticidal properties [13], and streptol-glucoside is a potent herbicide [14]. The bacterial symbionts are passed on to the next host generation via the seed [11, 15], but how the symbiotic bacteria reliably colonize seedlings in the presence of a complex soil and seed microbiome remains unknown.

The wild yam *Dioscorea sansibarensis* forms a permanent association with the bacteria *Orrella dioscoreae*, and offers an experimentally tractable system of heritable leaf symbiosis [16, 17]. The interaction most obviously takes place in a prominent gland at the tip of the leaves. We recently demonstrated that *O. dioscoreae* is vertically transmitted through vegetative propagules, called bulbils, and therefore present throughout the entire lifecycle of the plant [18]. Co-phylogenetic analyses indicate that the transmission route is not entirely closed, with horizontal transmission or host-switching also occurring in the wild [19]. However, the *D. sansibarensis–O. dioscoreae* symbiosis is ubiquitous in nature, implying the existence of mechanisms to enforce specificity [19]. In the laboratory, the plant and the bacteria can be grown separately, suggesting that this symbiosis is not obligate despite its high level of specificity in nature. Aposymbiotic plants develop normally *in vitro*, with fully formed leaf glands devoid of bacteria [20].

We demonstrated in a previous study that inoculation of aposymbiotic plants relies on colonization of the shoot apical bud, which contains the meristematic tissue at the origin of all above-ground organs throughout the life of the plant [18]. In this regard, plants face a drastically different situation from animals regarding vertical transmission of symbionts. Because the meristem links somatic and reproductive organs, plants cannot easily segregate symbionts between a germ line and a somatic population. Moreover, our previous studies showed that the apical bud remains physically open to the environment, creating opportunities for other microorganisms to establish in the plant at virtually any point during the growing phase [20]. What mechanisms evolved to enforce partner specificity in this context is unknown.

The order or timing of arrival of a species may also influence its success in establishing in a community, a phenomenon known as priority effect [21]. Hereditary symbionts may thus have an ecological advantage as they are already present at the earliest stages of a host’s lifecycle. The presence of *O. dioscoreae* within shoot buds may prevent strains with similar niche requirements from establishing in the plants via priority effects, for instance by pre-empting access to favored carbon or nitrogen sources. Priority effects may also be paired with other mechanisms such as interference competition, to ensure higher specificity. Type VI secretion systems (T6SSs) are known to have anti-microbial functions through delivery of effectors directly into targeted cells in a contact-dependent manner, and play a key role in microbe–microbe interactions [22, 23]. Indeed, the genome of *O. dioscoreae* harbors two T6SS gene clusters that are highly expressed in the leaf gland and conserved in all genomes of *O. dioscoreae* sequenced so far [19, 24]. Both gene clusters code for the 13 core proteins: TssABCEFGJKLM, VgrG, Hcp, and PAAR, as well as for accessory proteins, which usually play a role in regulation and modulation of the system assembling [25]. T6SSs are composed of three main parts, the integral membrane complex (TssJL and M), the baseplate (TssEFG and K), and the tail (Hcp, VgrG, PAAR, TssB and TssC). The tail is constituted of Hcp proteins polymerized into a stack of hexameric rings referred to as the inner tube, topped by the spike VgrG trimer and the PAAR protein [26–28]. The inner tube is surrounded by the sheath (TssBC). Upon contraction of the sheath, the inner tube and the spike are ejected into target cells, delivering the effector proteins [29–33]. In the contracted conformation the ClpV AAA+ ATPase is recruited to depolymerize the TssBC subunits, allowing recycling of the sheath [33–35]. The membrane complex is stable and may be used for multiple injections after reassembly [36]. Inactivation of the ClpV ATPase prevents recycling of the TssB and TssC components, thus precluding re-assembly of the membrane complex after effector delivery and effectively lowering the activity of the T6SS [34, 36, 37]. The role of T6SS-mediated competition in niche monopolization has been demonstrated in various Gram-negative bacteria. In particular, T6SSs were first described in the pathogenic bacterium *V. cholerae* as essential for both colonization and virulence [22, 38]. Specifically, T6SS-mediated competition is crucial for *V. cholerae* to outcompete resident microbiota in the human gut during disease [39].

The aim of this study was to acquire a better understanding of the mechanisms underlying specificity in the *D. sansibarensis–O. dioscoreae* symbiosis. We show that despite a ubiquitous and highly specific association, leaf glands of aposymbiotic *D. sansibarensis* are open to benign colonization by unrelated bacterial species with similar niche requirements. Co-inoculation assays on aposymbiotic seedlings show that *O. dioscoreae* is highly competitive *in planta*, and that this competitiveness is dependent upon a functional T6SS.

## Materials and methods

### Plant culture and propagation

*D. sansibarensis* Pax plants were obtained from the greenhouse of the Botanical Garden at the University of Ghent (LM-UGent) in Ghent, Belgium. Chemicals and reagents were purchased from Merck unless otherwise indicated. Plants used throughout in experiments were maintained at the Laboratory of Plants Microbes and Environment Interactions (LIPME) in Castanet-Tolosan, France. Unless otherwise indicated, plants were grown in climate-controlled chambers at 28°C, 70% humidity and a light cycle of 16 h light (210 μmol/m^2^/s), 8 h dark. Unless otherwise indicated, plants were kept in standard potting soil (PROVEEN SB-2; Bas Van Buuren B.V., Holland).

### Bacterial strains, plasmids, and growth conditions

Bacterial strains and plasmids are listed in Table S1. Routine culture of *O. dioscoreae* strains was done at 28°C in Tryptic Soy Broth (TSB, Sigma) or Tryptic soy agar (TSA, Sigma) supplemented with 30 μg/ml of nalidixic acid, 50 μg/ml of kanamycin, or 20 μg/ml of gentamicin where appropriate. *Stenotrophomonas* sp., *Rhizobium* sp., and *Sphingomonas* sp. strains were grown in TSB medium at 28°C unless otherwise specified. *P. putida* and *Escherichia coli* strains were grown in LB medium with appropriate antibiotics, at 28°C and 37°C respectively. To selectively grow *O. dioscoreae* R-71412 and derivatives*,* AB minimal medium [40] was supplemented with 0.01% (w/v) yeast extract (ABY medium), 10 mM trisodium citrate (Sigma), nalidixic acid (30 μg/ ml), and gentamicin (20 μg/ ml). Pseudomonas Isolation Agar (PIA, Sigma) supplemented with 2% glycerol was used to grow *P. putida* selectively.

### Production of aposymbiotic plants

Aposymbiotic plants were produced from node cuttings treated with antibiotics as described in [18]. Briefly, nodes were dissected from growth chamber-grown plants, and surface-sterilized for 8 h in a solution of 3× Murashige Skoog medium (MS, Sigma M5524) supplemented with 5% of Plant Preservative Mixture (PPM, Plant Cell Technology, USA). Explants were then aseptically transferred to sterile six-well plates containing MS medium supplemented with sucrose 2% (Merck), myo-inositol 555 μM (Sigma), glycine 26.6 μM (Sigma), cysteine 16.5 μM (Sigma), nicotinic acid 4.06 μM (Sigma), pyridoxine 2.96 μM (Sigma), thiamine 1.88 μM (Sigma), PPM (0.2% w/v), and the antibiotics carbenicillin and cefotaxim (200 μg/ ml each). Explants were incubated in a growth chamber at 28°C under 16 h/8 h day/night cycle. The medium was replaced after 10 days. After 3 weeks of incubation, the explants were aseptically transferred to sterile Magenta boxes (model GA7, Magenta) containing MS medium as above without the carbenicillin and cefotaxim. Effectiveness of the treatment was tested for each explant by collecting the first two leaves, milling in 100 μl of 0.4% w/v NaCl using a Retsch MM400 bead mill (1 m, 30 Hz), and spreading the macerate on TSA medium (Sigma). The absence of visible microbial growth after 2 days of incubation at 28°C was taken as evidence of the aposymbiotic status of the plants.

### Isolation and identification of leaf gland bacteria

Aposymbiotic plants were transferred from closed, sterile containers to open pots in the greenhouse (25°C, 16 h light/8 h dark, 60% humidity) for at least 8 weeks. Leaf acumens were dissected and surface-sterilized (5 m in 70% ethanol, sterile distilled water wash, 5 m in 1.6% sodium hypochlorite, three washes in sterile NaCl 0.4%). Samples were homogenized as described above and plated on TSA medium. Bacterial colonies were isolated on TSA. Identification was done by 16S rRNA gene sequencing using Sanger sequencing and PCR primers 27f and 1492r [41].

### Inoculations of *D. sansibarensis* aposymbiotic plant with bacteria

Overnight bacterial cultures were grown in LB or TSB as appropriate, and harvested by centrifugation at 7000 g for 5 m. Cell pellets were washed twice in sterile 0.4% NaCl. Bacterial suspensions were normalized to OD_600 nm_ = 0.2, corresponding to ~0.2 x 10^9^ CFU/ ml for *O. dioscoreae* and *P. putida* strains. Prior to inoculation, plants were moved to sterile Microbox containers (SacO2, Belgium) containing 50 ml of MS medium. Plants were inoculated by depositing 2 μl of the bacterial suspension directly onto the apical bud. Inoculated plants were grown at 28°C with a 16 h light/8 h dark cycle for 28 days. When applicable, a second inoculation was done a week later as described above. Colonization was evaluated by spreading serial dilutions of the milled and weighted leaf glands onto appropriate agar medium.

### Detection of bacteria in the second generation of inoculated plants

Previously inoculated plants were transferred from closed containers to open pots in growth chambers (25°C, 16 h light/8 hours dark, 70% humidity) until the end of the growing season. Bulbils were harvested and stored in a dry place until spontaneous sprouting. Sprouting bulbils were laid on a moist sand substrate and transferred to a growth chamber with high hygrometry (25°C, 16 h light/8 h dark, 90% hygrometry). Plantlets with at least two leaves were transferred to lower hygrometry (25°C, 16 h light/8 h dark, 70% hygrometry). Two leaf acumens per plant were tested for the presence of the bacteria. Leaf acumens were dissected and surface-sterilized as described above, and the presence and identity of bacteria were tested as described above.

### Observation of bacteria in leaf glands by fluorescence microscopy

Leaf glands were dissected with sterile scissors 8 weeks after inoculation and fixed in 0.3% paraformaldehyde in 0.1 M pH 7 potassium phosphate buffer for two hours at room temperature under vacuum. Samples were rinsed twice with potassium phosphate buffer (0.1 M, pH 7). 100 μm-thick sections were prepared using a vibrating microtome (Leica VT1000S) after embedding in 5% low melting agarose (Nusieve). Sections were observed using an epifluorescence microscope (Zeiss Axioplan2, excitation 475–40 nm and emission 530–50 nm). Images were processed using the ImageJ software version 1.54. Observation of non-fluorescent bacteria was done by collecting leaf glands 5 weeks post inoculation. Samples were prepared by sectioning with a razor blade, followed by staining with SYTO 9 (Thermo Fisher) and visualization using confocal microscope (Leica SP7). Images were processed using Leica LasX software.

### Bacterial genetics

T6SS deletion mutants were produced in an R-71412 background by homologous recombination as previously described [42, 43]. Briefly, the flanking regions of the gene to delete were amplified by PCR using specific primers (Table S2). The pSNW2 plasmid DNA was digested by XmaI (New England Biolabs) and gene fragments were assembled using the Pro Ligation-free cloning kit (Applied Biological Materials, Richmond, BC, Canada). All constructs were verified by whole-plasmid sequencing using ONT Nanopore sequencing [44]. *Orrella dioscoreae* R-71412 was electroporated with plasmid DNA as previously described, and kanamycin-resistant clones were selected [18]. Plasmid pQURE6 harboring a I-SceI nuclease gene was introduced into merodiploid clones by triparental mating. Colonies with gene replacement events were screened for loss of kanamycin resistance and by PCR. The double mutant was produced by deletion of ODI_R0808 in the previously obtained ODI_R3997 deletion mutant. The genome sequences of all mutant strains were obtained by Oxford Nanopore sequencing using R10.4 chemistry on an ONT P2 solo instrument.

For genetic complementation, gene fragments were amplified by PCR from *O. dioscoreae* R-71412 genomic DNA using specific primers (Table S2). Fragments were cloned into plasmid pSEVA2313 by restriction and ligation. *Escherichia coli* TOP10 was transformed by electroporation, plasmids were isolated from selected clones and validated by sequencing as above. Plasmid DNA was introduced into *O. dioscoreae* strains as above. Transformants were selected on TSA medium supplemented with kanamycin (50 mg/L).

### Bacterial phenotyping

Phenotyping Microarray (PM) plates were purchased from Biolog (USA). Bacteria were grown on solid media containing appropriate antibiotics and resuspended in sterile distilled water as per the manufacturer’s recommendation. Trisodium citrate was added to wells of the PM03 plate to 0.5% w/v final concentration. NADH oxidation was measured every 15 m for 120 h using an OmniLog instrument. Data were analyzed with the opm R package [45]. For quantitative comparison of *O. dioscoreae* R-71412 and *P. putida* KT2440*::gfp* metabolism on select carbon sources, custom Biolog plates were prepared with ABY medium supplemented with either trisodium citrate, L-malate, sodium succinate, fumarate or sodium pyruvate to 10 mM final concentration. NADH oxidation was measured as above. Data were analyzed on R using the Growthcurver package [46]. Optimal pH range for growth was tested in LB medium supplemented with 10 mM of trisodium citrate adjusted to pH = 5, 6, 7, or 8 and buffered with 100 mM of MES, MOPS or EPPS buffer as appropriate; nonadjusted (pH 7) LB-trisodium citrate medium was used as control. Cultures were inoculated from overnight cultures in TSB, diluted at 1:100. Bacterial growth was monitored by OD_600_ readings every 30 min for 48 h, using BMG FLUOstar Omega microplate reader. The growth rate *r* was calculated on R using the Growthcurver package [46].

### *In vitro* bacterial competition

LB or ABCY media were inoculated with a suspension of cells of *O. dioscoreae* mCherry-tagged strain R-71417 and/or with *P. putida* KT2440::*gfp* strain suspended in sterile distilled water at OD_600nm_ = 0.02. Cultures were inoculated with both strains at the same time. As a control for single strain growth, fresh media were inoculated in parallel using each of the same cell suspensions. Growth of *O. dioscoreae* R-71417 and *P. putida* KT2440::*gfp* were estimated by fluorescence measurement, respectively with mCherry (544 nm/590 nm) and GFP (485 nm/520 nm) filters in a BMG FLUOstar Omega microplate reader for 24 h with a measure taken every hour. Growth rate *r* and carrying capacity *K* were calculated from fluorescence intensity data using the Growthcurver R package [46]. Statistical analysis was conducted in R v4.1 [47].

### *In vitro* contact-dependent competition assay

*Orrella dioscoreae* R-71412, T6SS mutants and complemented strains, as well as fluorescent *P. putida* KT2440::*gfp* were grown overnight in TSB with appropriate antibiotics at 28°C. Cells were washed in a sterile solution of NaCl 0.4% w/v and density was normalized to OD_600 nm_ = 0.5. Then 10 μl of each sample were spotted onto a 96-well plate (Nunc FluoroNunc) filled with 180 μl of solid growth medium (TSA). Fluorescence was measured for 24 h at 28°C, in a BMG FLUOstar Omega microplate reader (set for spiral top reading, 30 reading points, adjusted with 90% of a 900 arbitrary fluorescence unit gain, filters: excitation 485–12 nm and emission 520–20 nm).

Alternatively, cultures of *O. dioscoreae* and *P. putida* or *Stenotrophomonas* sp. R-67087 strains were prepared as above and concentrated in sterile 0.4% NaCl solution to OD_600_ = 50 for *O. dioscoreae* strains and OD_600_ = 10 for *Stenotrophomonas* sp. and *P. putida*. Then, 5 μl of each sample were spotted onto pre-warmed TSA. After 2, 4, or 6 h at 28°C, cells were resuspended in 1 ml of NaCl 0.4% (w/v), serially diluted and plated on PIA and ABCY with appropriate antibiotics to estimate the respective number of *O. dioscoreae* and *P. putida* or *Stenotrophomonas* sp. colony-forming units (CFU).

### Ultra-high-performance liquid chromatography-high-resolution mass spectrometry analysis

Leaf acumens from aposymbiotic plants inoculated with *O. dioscoreae* R-71412 (6 plants, Sym1-Sym6) or with a solution of NaCl 0.4% (w/v; 4 plants, Apo1-Apo4) were dissected, and immediately frozen in liquid nitrogen. One leaf acumen was randomly chosen for each individual. Samples were milled using a Retsch MM400 bead mill (30 s, 30 Hz), and stored at −80°C until extraction. Samples were extracted with a mixture of methanol: water 80: 20, with a proportion of 1 ml of solvent per 100 mg of sample. Samples were dried and extracts were accurately weighed prior to diluting in solvent. Ultra-high-performance liquid chromatography-high-resolution MS (UHPLC-HRMS) analyses were performed on a Q Exactive Plus quadrupole (Orbitrap) mass spectrometer, equipped with a heated electrospray probe (HESI II) coupled to a U-HPLC Ultimate 3000 RSLC system (Thermo Fisher Scientific, Hemel Hempstead, UK). Separation was done on a Luna Omega Polar C18 column (150 mm × 2.1 mm i.d., 1.6 μm, Phenomenex, Sartrouville, France) equipped with a guard column. The mobile phase A (MPA) was water with 0.05% formic acid (FA), and the mobile phase B (MPB) was acetonitrile with 0.05% FA. The solvent gradient was 0 min, 100% MPA; 1 min, 100% MPA; 22 min, 100% MPB; 25 min, 100% MPB; 25.5 min, 100% MPA; and 28 min, 100% MPA. The flow rate was 0.3 ml/min, the column temperature was set to 40°C, the autosampler temperature was set to 5°C, and the injection volume was fixed to 5 μl. Mass detection was performed in positive ionization (PI) mode at resolution 35 000 power [full width at half-maximum (FWHM) at 400 m/z] for MS1 and 17 500 for MS2 with an automatic gain control (AGC) target of 1 × 106 for full scan MS1 and 1 × 105 for MS2. Ionization spray voltages were set to 3.5 kV, and the capillary temperature was kept at 256°C. The mass scanning range was m/z 100–1500. Each full MS scan was followed by data-dependent acquisition of MS/MS spectra for the six most intense ions using stepped normalized collision energy of 20, 40, and 60 eV. Raw data were processed with MS-DIAL version 4.70 for mass signal extraction between 100 and 1500 Da [48]. MS1 and MS2 tolerance were set to 0.01 and 0.025 Da in the centroid mode. The optimized detection threshold was set to 5 × 10^5^ concerning MS1 and 10 for MS2. Peaks were aligned on a QC reference file with a retention time tolerance of 0.15 m and a mass tolerance of 0.015 Da. Peak annotation was performed with an in-house database built on an MS-FINDER model [49]. MS-DIAL data were then cleaned with the MS-CleanR workflow by selecting all filters with a minimum blank ratio set to 0.8, a maximum relative standard deviation (RSD) set to 30, and a relative mass defect (RMD) ranging from 50 to 3.000. The maximum mass difference for feature relationships detection was set to 0.005 Da and the maximum RT difference to 0.025 min. Pearson correlation links were considered with correlation ≥0.8 and statistically significant with α = 0.05. Two peaks were kept in each cluster, viz., the most intense, and the most connected. The kept features (m/z × RT pairs) were annotated with MS-FINDER version 3.52. The MS1 and MS2 tolerances were, respectively, set to 10 and 20 ppm. Formula finders were only processed with C, H, O, N, and S atoms. Databases (DBs) based on *Dioscorea* (genus), *Alcaligenaceae* (family) were constituted with the dictionary of natural products (DNP, CRC press, DNP on DVD v. 28.2). The internal generic DBs from MS-FINDER used were KNApSAcK, PlantCyc, NANPDB, UNPD, COCONUT, and CheBI. Data were normalized per sample based on the sum of all peak areas. Statistical analyses were done using the R software and standard packages.

## Results

### Aposymbiotic *D. sansibarensis* are open to colonization by bacteria other than *O. dioscoreae*

**Figure 1:**
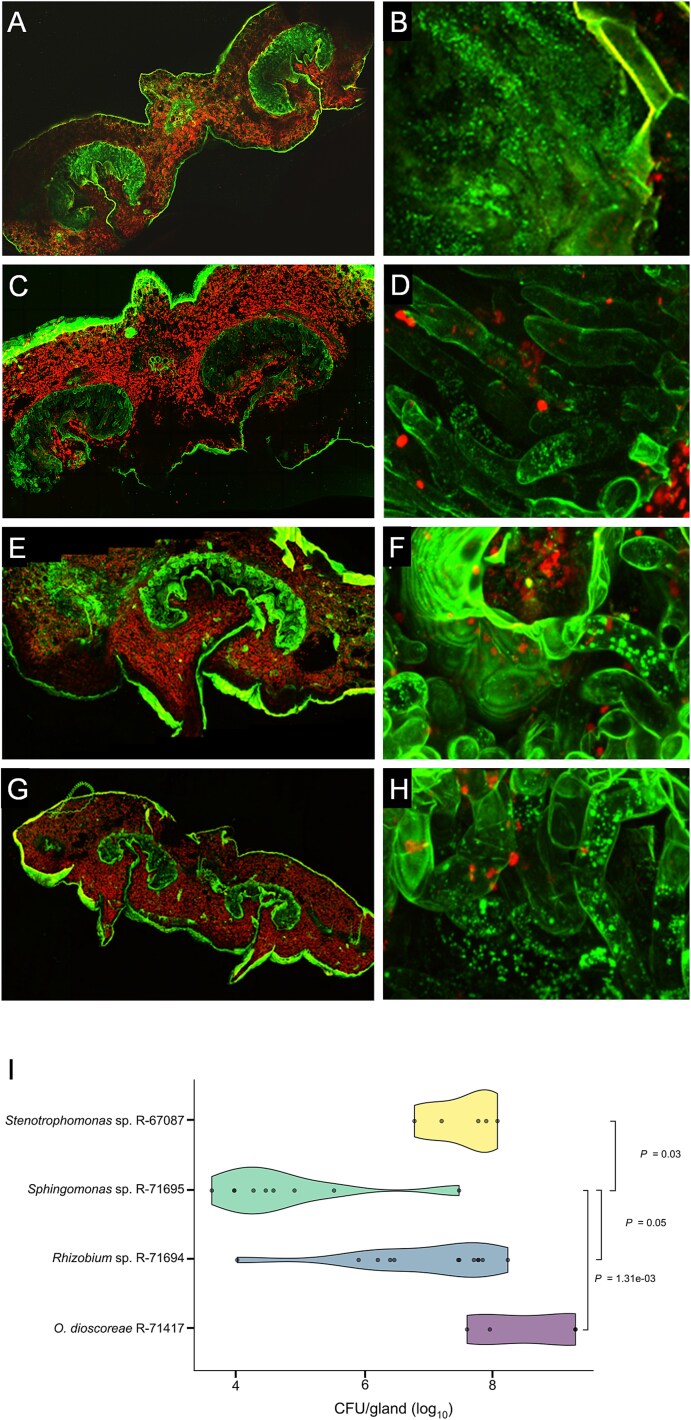
Nonsymbiotic bacteria effectively colonize *D. sansibarensis* leaf glands. Confocal images of cross sections of *D. sansibarensis* leaf glands colonized by taxonomically diverse bacterial strains after artificial inoculation. *D. sansibarensis* leaf glands colonized by (A) *O. dioscoreae* R-71412, (C) *Rhizobium* sp. R-71694, (E) *Stenotrophomonas* sp. R-67087, and (G) *Sphingomonas* sp. R-71695. Details of trichomes surrounded by mucus and green foci corresponding to stained bacteria (B) *O. dioscoreae*, (D) *Rhizobium* sp., (F) *Stenotrophomonas* sp., and (H) *Sphingomonas* sp. Bacteria were stained with SYTO 9 and autofluorescence was used to visualize plant cells. I. Bacterial titers inside surface-sterilized leaf glands of *D. sansibarensis* inoculated with the same strains as above (CFU/leaf gland). Bars indicate statistically significant differences between groups according to a Kruskal-Wallis rank sum with Dunn’s post-hoc test and Bonferroni correction for multiple testing. For clarity, only pairwise comparisons with *P*-value adjusted <0.05 are shown.

We reported previously that aposymbiotic *D. sansibarensis* plants were amenable to colonization by exogenously applied *O. dioscoreae*, suggesting that aposymbiotic plants remained receptive to bacteria during vegetative growth. To test whether this applied to bacteria other than *O. dioscoreae*, we produced bacteria-free plants by antibiotic treatment of explants. After ~2 months in sterile cultures, plants were moved to open pots for the rest of the growing season. The leaf glands of several of those aposymbiotic plants became spontaneously colonized with bacteria unrelated taxonomically to *O. dioscoreae* (Table 1). We selected three isolates belonging to the genera *Stenotrophomonas* sp. (R-67087)*, Rhizobium* sp. (R-71694), and *Sphingomonas* sp. (R-71695) for further study. Artificial inoculations of aposymbiotic plants showed that these three strains occupy the lumen of the leaf glands, similar to *O. dioscoreae* R-71412 (Fig. 1A–H). Strains *Stenotrophomonas* sp. (R-67087) and *Rhizobium* sp. (R-71694) reached similar population densities within the leaf gland as *O. dioscoreae* (averages ranging from 0.4 to 10.3 x 10^8^ cfu/leaf acumen, *P* > .05) (Fig. 1). *Sphingomonas* sp. (R-71695) also colonized leaf glands, but yielded much lower average densities at 0.03 x 10^8^ CFU/leaf gland (Fig. 1).

**Table 1:** Identification of isolates from glands of aposymbiotic *Dioscorea sansibarensis.*

Plants colonized	Best BLAST hit		Identity (%)
5	*Rhizobium radiobacter* ATCC 19358		99.7
1	*Cupriavidus plantarum* ASC-64		99.4
1	*Variovorax* sp. PMC12		100.0
1	*Pantoea* sp. ND03		99.9
1	*Pseudomonas atacamensis* M7D1		99.8
1	*Enterobacter soli* ATCC BAA-2102		99.7
1	*Agrobacterium* sp. K599		100.0
2	*Stenotrophomonas indicatrix* WS40		100.0
1	*Pseudomonas fortuita* GMI12077		100.0
2	*Pseudomonas lactis* DSM 29167		100.0
1	*Pseudomonas citronellolis* NBRC 103043		100.0
1	*Pseudomonas neuropathica* P155		99.9
1	*Pseudomonas paralactis* DSM 29164		99.5
1	*Cupriavidus campinensis* WS2		99.7
1	*Sphingomonas* sp. 1429(T)		97.6

### Characterization of the metabolic niche of *O. dioscoreae*

The diverse taxonomic range isolates from aposymbiotic leaf glands prompted us to test whether bacteria with similar physiological properties to *O. dioscoreae* might be able to colonize aposymbiotic *D. sansibarensis*. *O. dioscoreae* grows aerobically, requires a range of temperatures for growth of 15–40°C, and pH slightly acidic to neutral (Fig. S1). We screened a library of compounds to get a comprehensive overview of potential nutrients available for growth of *O. dioscoreae*. Of the 190 carbon sources tested, only 29 were oxidized by cultures of *O. dioscoreae* after 72 h (Table S3). The oxidation of citrate, L-malate, succinate, and fumarate indicated that assimilation pathways converge towards the tricarboxylic acid cycle (TCA). Hexose sugars were not utilized by *O. dioscoreae*, and gluconate supported growth for some, but not all, strains [17]. We also screened 95 compounds as potential sources of nitrogen (Table S3). In particular, ammonia, urea, and the amino acids L-Ala, L-Asn, L-Asp, L-Glu, L-Gln, Gly, L-His, L-Ile, L-Leu, L-Pro, L-Ser, L-Met, D-Ala, D-Asp, D-Glu, and D-Ser supported cellular activity as nitrogen sources. Metabolomics analysis of symbiotic vs. aposymbiotic glands show distinct metabolic profiles (Figs. S2 and S3), with differentially abundant features dominated by fatty acids. These potentially correspond to plant vs bacterial membrane lipids that are expected to vary as symbiotic sample types contain large amounts of bacteria absent in aposymbiotic samples. The data also confirmed that malate, citrate, aspartate, and glutamate were abundant in whole leaf glands, with citrate and malate being the fourth and 14th most abundant ions overall (Table 2). Malate was also significantly enriched by nearly two-fold in aposymbiotic vs. symbiotic leaf glands (Student t-test *P* = .012). Concentrations of citrate were not significantly different, but tended to be more abundant in extracts of aposymbiotic leaf glands, (1.82× *P* = .087) (Table 2). We were unable to detect other intermediates of the TCA in our samples, but the fact that citric acid and malic acid were abundant and slightly depleted within symbiotic leaf glands suggests that these compounds may be metabolized by the bacteria within the leaf gland.

**Table 2:** Detection of metabolites supporting the growth of *O. dioscoreae* in symbiotic and aposymbiotic leaf glands of *Dioscorea sansibarensis*. Average peak intensity of selected metabolites detected in the symbiotic and aposymbiotic leaf glands of *D. sansibarensis*. Statistical significance of the fold change was calculated by Student’s T-test. Standard deviations are indicated for each average intensity value. *^a^* Feature ID corresponds to the field “Alignment ID” of Table S4 and S5. *^b^* Peak intensities are reported normalized and with background subtracted (mean value from blank samples). Negative values correspond to features that have higher average peak intensities in injection blanks. *^c^* Mean peak intensity across all samples (blanks excluded). Rank is based on mean peak intensity of all features, with lower ranks indicating higher average abundances.

Metabolite	Feature ID*^a^*	Peak Intensity			Fold change	Student’s T-test *P* value
		Mean (rank)*^c^*	Aposymbiotic	Symbiotic		
Citrate	162_C18neg	5442.9 (4/1367)	7310.6 ± 3646.7	4022.1 ± 2342.1	1.82	0.087
Gluconate	175_C18neg	294.1 (102/1367)	330.2 ± 124.0	233.7 ± 140.2	1.42	0.133
Aspartic acid	63_C18neg	389.8 (84/1367)	487.1 ± 169.6	299.3 ± 123.0	1.63	0.056
L-pyroglutamic acid*^b^*	51_C18neg	29.5 (812/1367)	−34.0 ± 3.2	−39.6 ± 9.6	0.86	0.091
Malate	66_C18neg	2471.5 (14/1367)	3520.5 ± 909.2	1760.7 ± 534.4	2.00	0.012
L-Glutamine	83_C18neg	226.7 (130/1367)	145.2 ± 71.42	249.6 ± 76.6	0.58	0.041
L-glutamic acid	84_C18neg	461.4 (76/1367)	391.4 ± 54.89	427.4 ± 219.5	0.92	0.355
Galactonic acid gamma-lactone*^b^*	323_C18pos	23.8 (927/1367)	−35.9 ± 0.91	−3.7 ± 19.3	9.69	0.002
L-Serine	4_C18neg	483.2 (72/1367)	875.2 ± 370.06	206.8 ± 140.1	4.23	0.016

### Metabolic niche overlap between *P. putida* and *O. dioscoreae*

**Figure 2:**
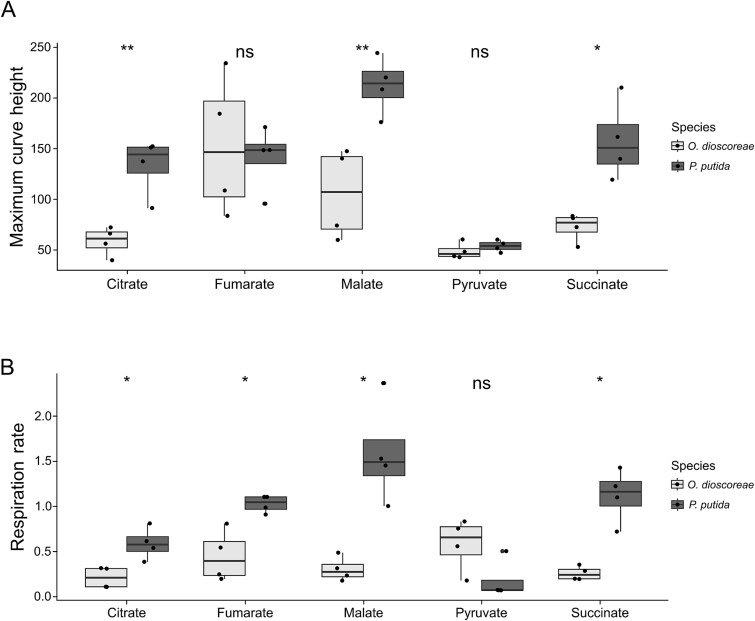
Utilization of substrates found in leaf glands by *P. putida* and *O. dioscoreae*. Substrate oxidation by *O. dioscoreae* R-71412 and *P. putida* KT2440*::gfp* was monitored on ABY minimal medium supplemented with trisodium citrate (ABCY), fumarate, L-malate, sodium pyruvate or sodium succinate for 48 h. (A) Maximum Curve height (absorbance) and (B) Respiration rates were computed from curves modeled with R package Growthcurver. Data corresponding to *P. putida* are shown in dark grey, and *O. dioscoreae* in light grey. Statistical significance between each strain for each carbon source was calculated using a one-way ANOVA (significance levels: Ns *P* > .05; ^*^P <= .05; ^**^P <= .01; ^***^P value <= .001; ^****^P <= .0001).

We hypothesized that the ability to utilize metabolites found in the leaf gland might explain the ability of bacteria to colonize *D. sansibarensis*. However, strains from our collection of isolates (Table 1), might share underlying adaptations necessary for an endophytic lifestyle inside *D. sansibarensis*, such as the ability to circumvent host immunity or to avoid competition with *O. dioscoreae*. We therefore searched the available literature for bacterial strains capable of utilizing the identified carbon sources of *O. dioscoreae*. *Pseudomonas putida* KT2440 is a well-described model organism capable of growth on many organic acids, and is derived from *P. putida* mt-2 originally isolated from soil [50, 51]. The metabolic requirements of strain KT2440 are well-documented and comprise those of *O. dioscoreae* [52–56]. First, we confirmed that a GFP-tagged derivative of *P. putida* KT2440 could utilize a majority of the carbon (22/29) and nitrogen sources (41/41) that support growth of *O. dioscoreae* using Biolog phenotype microarrays (Table S3). We next measured the respiration of *O. dioscoreae* and *P. putida* KT2440::*gfp* on five substrates likely to occur within the plant environment: citrate, L-malate, succinate, fumarate, and pyruvate. Cultures of *P. putida* KT2440::*gfp* showed significantly higher yield (Fig. 2A) and respiration rate (Fig. 2B) with L-malate, citrate, and succinate than *O. dioscoreae*. We detected no differences between the two strains regarding the oxidation of fumarate or pyruvate. Generally faster respiration rates and higher final yields indicate that *P. putida* is a strong competitor against *O. dioscoreae* for all potential substrates found in the leaf gland.

**Figure 3:**
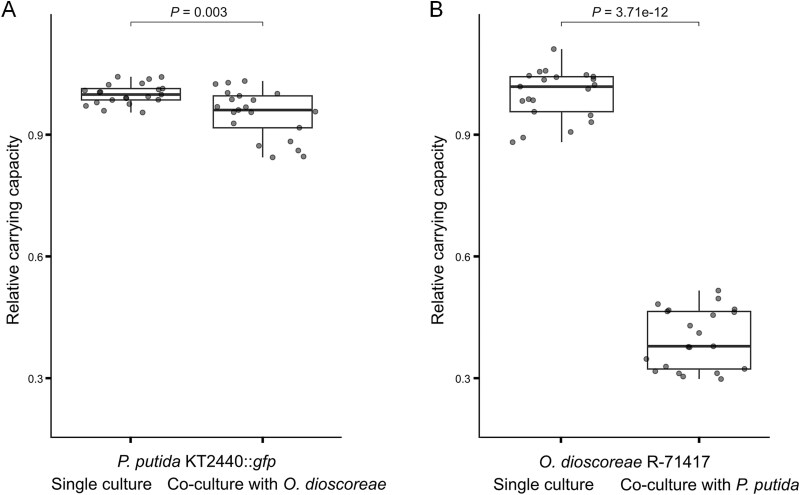
*In vitro* competition between *P. putida* and *O. dioscoreae*. Cultures in LB medium in microtiter plates were inoculated with strains *P. putida* KT2440*::gfp* and mCherrry–tagged *O. dioscoreae* R-71417 together or separately. GFP- and mCherry-specific fluorescence was used to monitor the growth of each strain and derive growth characteristics. Values were normalized to the average of values in single culture. (A) Carrying capacity of strain *P. putida* KT2440*::gfp* inferred from GFP-specific fluorescence in single (left) or in co-culture with *O. dioscoreae* R-71417. (B) Carrying capacity of strain *O. dioscoreae* R-71417 inferred from mCherry-specific fluorescence in single (left) or co-culture with *P. putida* KT2440*::gfp*. Horizontal bars indicate *P-*values (Wilcoxon rank sum test). Data shown were collected from three independent experiments.

To test whether both strains also compete for the same resources in a more complex environment, we monitored growth of *O. dioscoreae* R-71417 and *P. putida* KT2440::*gfp* in single and co-cultures. The carrying capacity of *P. putida* was significantly decreased in the presence of *O. dioscoreae* in LB medium, although by a mere 5% (Fig. 3A). However, the carrying capacity of *O. dioscoreae* R-71417 was severely impacted when co-cultured with *P. putida* KT2440::*gfp*, with a decrease of 62% in LB (Fig. 3B) and 77% in ABCY medium (Fig. S4A). In contrast, the carrying capacity of *P. putida* was significantly increased by 26% when grown in the presence of *O. dioscoreae* in ABCY medium (Fig. S4A). This correlates with a 42% decrease in growth rate (Fig. S4B), suggesting a metabolic shift towards more efficient resource utilization. Together, these results indicate that *P. putida* and *O. dioscoreae* compete for resources in liquid cultures. In addition, this suggests that *P. putida* KT2440::*gfp* adapts its growth strategy depending on the quality of the environment to consistently outgrow *O. dioscoreae*.

### Effective colonization of *D. sansibarensis* leaf glands by *P. putida*

**Figure 4:**
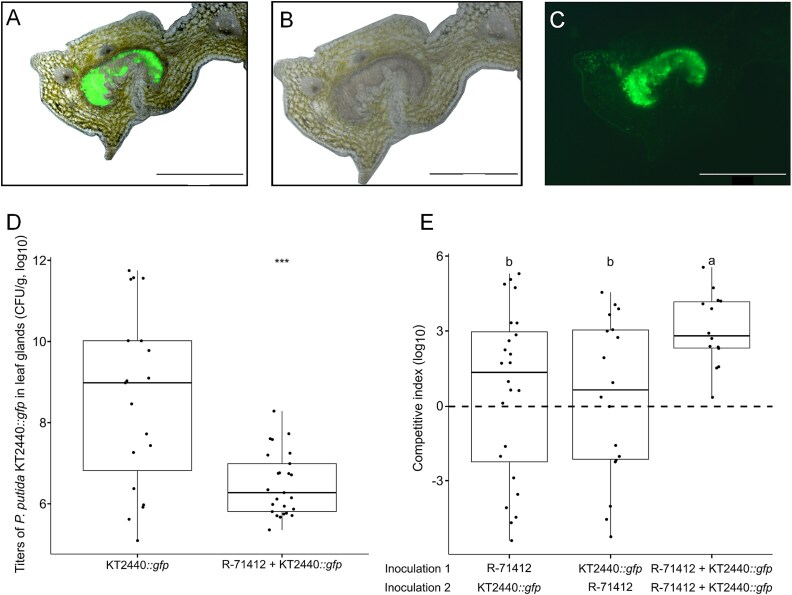
*P. putida* colonization of *D. sansibarensis* leaf glands. (A) Cross-section of a *D. sansibarensis* acumen colonized by *P. putida* KT2440**::*gfp* after artificial inoculation. Merged image of light microscopy (B) and epifluorescence (C) observations. Scale bar = 500 μm. (D) Quantification of *P. putida* KT2440**::*gfp* in leaf glands of *D. sansibarensis* after single inoculation or co-inoculation with *O. dioscoreae* R-71412 in equal ratios. Student’s t-test significance levels: Ns *P* > .05; ^*^  *P* < .05; ^**^P < .01; ^***^P < .001; ^****^P < .0001. (E) Competitive index of *O. dioscoreae* R-71412 in successive co-inoculations with *P. putida* KT2440::*gfp*. Aposymbiotic plants were inoculated first with either *O. dioscoreae* R-71412 or *P. putida* KT2440**::*gfp*, or with the same strain twice. Competitive index was calculated as the log10 of the leaf gland colonization (CFU per g) by *O. dioscoreae* R-71412 over that of *P. putida* KT2440::*gfp*. Horizontal lines represent the median. Different letters indicate statistically significant differences between groups according to ANOVA with Tukey post-hoc test.

To test whether *P. putida* KT2440 could establish stable colonization in *D. sansibarensis*, we inoculated aposymbiotic plantlets with ~4 × 10^5^ CFU of *P. putida* KT2440::*gfp*. We detected *P. putida* KT2440::*gfp* within the glands of all plants colonized with the strain, but in none of the mock-inoculated controls. Epifluorescence imaging of leaf tip sections harvested 2 months after inoculation further confirmed that cells of *P. putida* KT2440::*gfp* occupied the lumen of the leaf glands (Fig. 4A–C). Leaf glands of plants inoculated with *P. putida* KT2440::*gfp* contained an average of 10^9^ CFU/g of fresh tissue (Fig. 4D), whereas macerates of lamina of surface-sterilized leaves did not yield colonies upon plating on nonselective TSA growth medium. This is similar to the colonization levels of wild-type *O. dioscoreae* in leaf glands of *D. sansibarensis* (Fig. 1).

### Competitive advantage of *O. dioscoreae* in the leaf gland

Because colonization of leaf glands did not seem to be specific to *O. dioscoreae*, we wondered whether better niche adaptation of *O. dioscoreae*, or competitiveness, might account for specificity in nature. To test this, we co-inoculated aposymbiotic plants with cell suspensions of *P. putida* KT2440::*gfp* alone or mixed with *O. dioscoreae*. The presence of *O. dioscoreae* reduced the average titer of *P. putida* KT2440::*gfp* in the leaf gland from 9.15×10^10^ to 1.67×10^7^ CFU/g (Fig. 4D). Although co-culture experiments in liquid media may allow for the detection of nutrient-based competition, they fail to account for several factors that could be relevant inside the host. Niche conditioning by *O. dioscoreae* might explain the lower rates of growth of commensal strains in the leaf gland in presence of *O. dioscoreae* (e.g. by depleting nutrients, saturating favored spatial niches, secreting anti-microbial compounds, or inducing specific plant defenses). Manipulating the order of inoculation between the strains would thus be expected to exacerbate the differences. To test this, we inoculated the two strains in varying order: *O. dioscoreae* first, *P. putida* KT2440::*gfp* first or both simultaneously. Co-inoculations always resulted in a significant competitive advantage of *O. dioscoreae* (Fig. 4E). However, we did not observe a significant effect of the order of arrival on the competitive index of either strain, indicating that niche conditioning or priority effects do not account for the majority of the competitive advantage of *O. dioscoreae* in the leaf gland.

### Competitive advantage of *O. dioscoreae* is mediated by T6SSs

**Figure 5:**
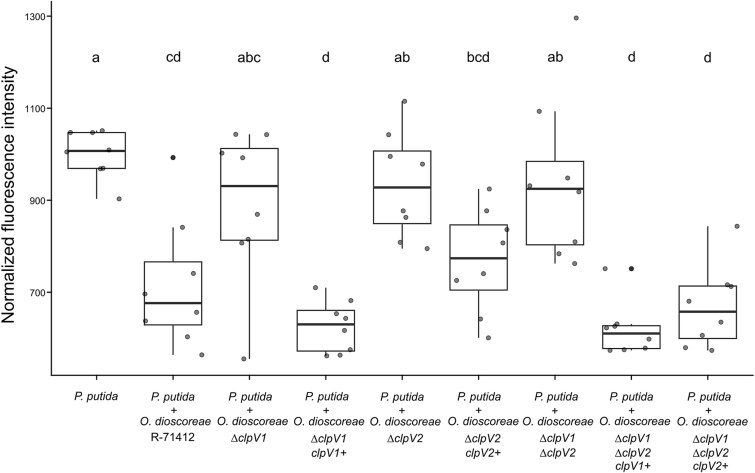
Contact-dependent competition between *O. dioscoreae* and *P. putida*. Fluorescence intensity of cultures of *P. putida* KT2440**::*gfp* after 4 h of growth alone or in contact with *O. dioscoreae* strains. *O. dioscoreae* T6SS mutants *∆clpV1*, *∆clpV2*, and *∆clpV1∆clpV2* and complemented strains *∆clpV1/clpV1+*, *∆clpV2/clpV2+*, *∆clpV1∆clpV2/clpV1+*, and *∆clpV1∆clpV2/clpV2+* were tested. Different letters indicate statistically significant differences between groups according to ANOVA with Tukey post-hoc test (significance threshold *P* = 0.05). Results shown here are from one of three independent experiments showing similar results.

The genome of *O. dioscoreae* encodes two T6SSs that belong to two different phylogenetic subclasses, and possess different arsenals of effectors (Fig. S5). Both are expressed *in planta* [24], and we reasoned that anti-microbial effectors secreted by these T6SS may give a competitive advantage to *O. dioscoreae*. We tested contact-dependent growth inhibition of *P. putida* KT2440::*gfp* by *O. dioscoreae* in a microtiter plate assay. We observed a significant decrease of GFP-specific fluorescence by 29% in the presence of *O. dioscoreae* R-71412 (Fig. 5). This effect was reduced (6–11% reduction, *P* > .05) in the presence of strains harboring mutations in either or both of the T6SS *clpV* genes, although *O. dioscoreae* mutant strains had similar growth characteristics (Fig. S6). Complementation of the single and double mutant with copies of *clpV1* or *clpV2 in trans* restored wild-type levels of competition against *P. putida* (Fig. 5).

To confirm that contact-dependent killing is responsible for the reduction in GFP-specific fluorescence in the microtiter plate assay and to gain more quantitative insights, we counted the number of CFU by dilution plating of both *P. putida* KT2440::*gfp* and *O. dioscoreae* strains after direct contact for 2, 4 or 6 h. We observed a reduction by an average of 6429-fold in the number of *P. putida* KT2440::*gfp* colonies upon co-culture with *O. dioscoreae* R-71412, but not with the *ΔclpV1ΔclpV2* mutant (1.2-fold decrease, Fig. S7). The effect was also pronounced upon contact with the *ΔclpV1* strain (9520-fold average reduction), but not with the *ΔclpV2* strain (nine-fold reduction, *P* > .05). This indicates that the contribution of T6SS-2 to the killing of *P. putida* is larger than that of T6SS-1. However, the significant reduction of *P. putida* CFU counts in competition with the *ΔclpV1ΔclpV2* vs. the *ΔclpV2* strain (7.58-fold, *P* < .05) indicates that, although minute, the contribution of T6SS-1 is still detectable in this assay.

**Figure 6:**
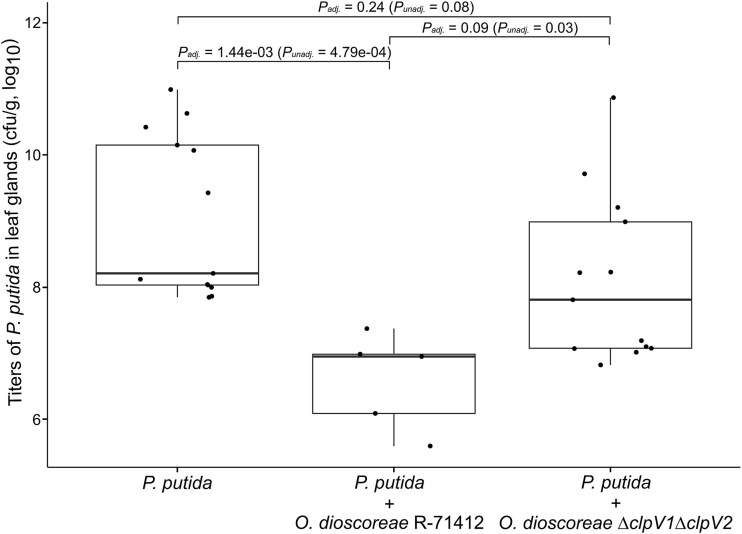
*In planta* competition between *P. putida* and strains of *O. dioscoreae* affected in type VI secretion. Aposymbiotic plants were inoculated with *P. putida* KT2440*::gfp* alone or in 1:1 ratio with *O. dioscoreae* strain R-71412, or strain *∆clpV1∆clpV2*. Freshly grown acumens were weighed and macerated. Serial dilutions were plated on selective media to quantify bacterial load. Horizontal bars indicate *P*-values between groups according to Dunn’s test. *P-*values adjusted for multiple testing with the Bonferroni method are shown, with unadjusted *P*-values in parentheses. Results shown here are from one of two independent experiments showing similar results.

We performed a co-inoculation experiment to test the contribution of T6SS to the competitive advantage of *O. dioscoreae in planta*. The T6SSs of *O. dioscoreae* did not affect the ability to colonize the leaf gland in single inoculations (Fig. S8). The presence of the wild-type strain of *O. dioscoreae* negatively impacted the colonization of *P. putida* KT2440::*gfp* by three orders of magnitude (1703-fold fewer CFU on average, *P* = 1.44x 10^−3^) (Fig. 6). In contrast, this negative effect was much alleviated when co-inoculating plants with the *O. dioscoreae ΔclpV1ΔclpV2* strain, with only a 2.39-fold reduction in the number of *P. putida* CFU (*P* = .24). Microscopy observations of leaf glands by epifluorescence further confirmed that wild-type *O. dioscoreae* R-71412 effectively blocked the colonization of leaf glands by *P. putida* KT2440*::gfp*, but *O. dioscoreae ΔclpV1ΔclpV2* did not (Fig. S9). Colonization of *D. sansibarensis* by *Stenotrophomonas* sp. R-67087 was also attenuated (23-fold, *P* = .04) when co-inoculated with *O. dioscoreae* R-71412, but not with *O. dioscoreae ΔclpV1ΔclpV2*, confirming the role of T6SS in antagonism against diverse taxa capable of colonizing the leaf gland (Fig. S10).

### Evidence of species-selective transmission

We tested whether *P. putida* could also be transmitted to the next generation of plants. To test this, we inoculated plants with *P. putida* KT2440::*gfp*. We collected bulbils produced by the plants at the end of the growing season and let them germinate after a storage period of 10 to 16 months. The plants produced on average 5.9 bulbils per plant, with a bulbil germination rate of 9.1% (Table 3). None of the daughter plants contained detectable *P. putida* in the leaf glands. As a control, we also inoculated plants with a 1:1 cell mixture of *P. putida* KT2440::*gfp* and *O. dioscoreae* R-71412. These produced 4.4 bulbils per plant on average, for a bulbil germination rate of 15.7%. Of the 109 daughter leaf glands tested only one contained both *P. putida* KT2440::*gfp* and *O. dioscoreae* (0.9% of the leaf glands), whereas we detected *O. dioscoreae* R-71412 in 69 samples (63.3%). The plants inoculated with *P. putida* KT2440*::gfp* and *O. dioscoreae ΔclpV1ΔclpV2* produced 11.86 bulbils by plant on average. None of the 26 leaf glands of the progeny contained *P. putida* but 18 leaf glands contained *O. dioscoreae* (69.2%). This confirmed that the T6SS is not relevant for the vertical transmission, even in presence of nonsymbiotic bacteria.

**Table 3:** Detection of bacteria in offspring of inoculated *Dioscorea sansibarensis.*

			Bacteria detected in leaf glands of offspring			
Inoculum	Average number of bulbils produced by plant	Sprouting efficacy (%)	*P. putida* only	*O. dioscoreae* only	*O. dioscoreae* and *P. putida*	No bacteria
***P. putida***	5.9	9.1	0	0	0	35
***P. putida* + *O. dioscoreae* R-71412**	4.4	15.7	0	68 (62.3%)	1 (0.9%)	40 (36.7%)
***P. putida* + *O. dioscoreae ΔclpV1ΔclpV2***	5.6	11.7	0	18 (69.2%)	0	8 (30.8%)

## Discussion

Heritable leaf symbioses are highly specific in nature: one plant species associates with only one bacterial species. At least in the case of the *D. sansibarensis* leaf symbiosis, this specificity is not due to an obligate interaction: aposymbiotic plants develop normally in a controlled environment, and bacteria can grow axenically as well [18]. Several recent studies have shown that free-living bacteria could effectively replace vertically transmitted insect symbionts, under some conditions. For example, the obligate symbiont *Sodalis pierantonius* of the grain weevil *Sitophilus zeamais* could be replaced by a free-living strain of *Sodalis praecaptivus* engineered to secrete aromatic amino acids [57]. Similarly, a free-living *E. coli* strain was successfully evolved in the laboratory and provided an effective replacement for the vertically transmitted *Pantoea* symbiont of *Plautia stali* stinkbugs upon acquisition of a mutation in global regulators controlling carbon utilization pathways [58]. In both of these examples, stable symbiont replacement occurred with closely related strains (same genus or family) after some genetic modification. Here, we show that a vertically transmitted plant symbiosis is promiscuous, with taxonomically diverse strains capable of stable colonization. This is in stark contrast to the 100% specificity for *O. dioscoreae* observed in field samples [19, 24].

Using metabolomics data from *D. sansibarensis* leaf glands and metabolic profiling of *O. dioscoreae*, we established the conditions likely encountered by the bacteria inside the plant. Conditions in the *D. sansibarensis* leaf glands are micro-oxic (De Meyer *et al.* 2019) *O. dioscoreae* grows best at neutral to slightly acidic pH. Additionally, *O. dioscoreae* putative high-affinity iron-acquisition systems are overexpressed *in planta* compared to axenic cultures [24]. This is perhaps indicative of depletion of iron in the leaf gland, which may be associated with plant immunity [59]. Furthermore, genes involved in oxidative stress responses (e.g. putative catalase and peroxidases) are overexpressed by the bacteria *in planta* and functions linked to resistance to oxidative stress are highly conserved in the core genomes of leaf symbionts [24, 60]. TCA cycle substrates could be commonly used as major carbon sources by leaf endosymbionts. Taken together, these data indicate that the conditions in the leaf glands may contribute to selecting bacterial specialists adapted for utilization of acids and tolerance to oxidative stress, but may not be otherwise particularly stringent.

*P. putida* KT2440 presents an overlapping metabolic profile to *O. dioscoreae* and is able to colonize the leaf glands of *D. sansibarensis* to high densities. *Pseudomonas putida* KT2440 derives from a soil isolate and is unlikely to be pre-adapted to an endophytic lifestyle. This suggests that once the plant is deprived of its symbiont the leaf gland becomes open to colonization by other bacterial strains. Moreover, *P. putida* is metabolically well adapted to inter-species competition [61, 62]. Our results show that *P. putida* KT2440*::gfp* consistently outcompeted *O. dioscoreae* in liquid cultures, adopting seemingly distinct strategies to maximize resource utilization. This is illustrated by the fact that upon entering co-culturing with *O. dioscoreae* in rich medium (LB), which offers a broad metabolic niche, *P. putida* increases growth rate at the cost of a lower carrying capacity (Fig. S4C). In contrast, ABCY medium offers a narrower metabolic niche, with citrate as the only abundant carbon and energy source. Upon competition with *O. dioscoreae*, the growth rate of *P. putida* in ABCY medium slows down, perhaps allowing for greater efficiency in resource utilization and maximizing carrying capacity (Fig. S4A and B). Despite this adaptive metabolic potential, *P. putida* is unable to outcompete *O. dioscoreae* in plant co-inoculation assays (Fig. 4).

In *D. sansibarensis* glands, bacteria are present to high titer up to 1×10^10^ CFU/g of tissue, at least an order of magnitude higher than what is achievable in liquid cultures. High cell-density and proximity may therefore allow for other mechanisms of competition, for example for contact-dependent competition. Experiments on solid media indeed showed that *O. dioscoreae* kills *P. putida* KT2440::*gfp* cells in a contact-dependent manner, and that this antagonistic activity is mediated by two T6SS gene clusters encoded in the genome of *O. dioscoreae.* Both T6SS-1 and T6SS-2 of *O. dioscoreae* LMG29303^T^ are overexpressed *in planta* and are conserved in all *O. dioscoreae* genomes, although the repertoire of predicted effectors vary between strains [19, 24]*.* The anti-microbial activity of T6SS and associated effectors is well-described, and contrasting T6SS effector sets may reflect varying pathogen or competitor pressures in natural populations of *O. dioscoreae* (reviewed in [35, 63]). Both *O. dioscoreae* T6SS clusters encode effector proteins with putative anti-microbial activities. The T6SS-1 cluster encodes a putative colicin (ODI_R3996) and a VgrG-family protein with a C-terminal Tle1 phospholipase domain (ODI_R3993). T6SS-2 encodes effectors containing Tle4 (ODI_R794), M15 metallopeptidase (ODI_R0790), and Tle1 (ODI_R0793) domains. Aside from a role in protecting the symbiotic niche, it is unclear whether *O. dioscoreae* also provides protection against bacterial or fungal pathogens, as has been demonstrated in other systems [64, 65]. Complete T6SS were only detected in the genomes of leaf symbionts of *Fadogia homblei* and *Vangueria pygmaea*, but not in other leaf symbionts of the *Caballeronia* clade [12]. How specificity is maintained in leaf symbioses in the absence of a T6SS remains unknown, but other secreted toxins might play a role.

Alone, antagonistic microbe–microbe interactions are unlikely to fully explain the absolute specificity between *D. sansibarensis* and *O. dioscoreae* observed in the wild. Type III secretion systems (T3SS) are often involved in associations with eukaryotic hosts through delivery of effectors into host cells [66]. These effectors may modulate immune pathways and play a key role in symbiotic partner recognition and specificity [67]. Genomes or metagenome-assembled genomes (MAGs) of *O. dioscoreae* isolated from leaf glands of *D. sansibarensis* lack a conserved T3SS, suggesting that this pathway is not essential for the association [19]. Although the genome of *O. dioscoreae* LMG 29303^T^ (the parental strain for all strains used in this study) does encode a minimal T3SS gene cluster, previous studies did not find evidence of expression *in vitro* or *in planta* [24]. The fact that *P. putida* readily colonized *D. sansibarensis* in the absence of a functional T3SS is further evidence that secretion of T3 effectors is not required for colonization. The absence of a phenotype in aposymbiotic plants suggests that symbiotic functions may be important for fitness in response to environmental or herbivory pressures. We are currently assessing the phenotypes of plants under an array of conditions. If the symbiosis is indeed essential for survival under natural conditions, vertical transmission, and partner-fidelity feedback may also contribute to specificity by culling plants with ineffective symbionts from the population [68, 69].

Despite *D. sansibarensis* being amenable to artificial inoculations, transmission of symbionts to bulbils appears more stringent. Transmission appears to be imperfect in our experiments, with symbiotic bacteria detected in only 62% of bulbils. This indicates that barriers to transmission exist. These barriers appear somewhat selective, with plants inoculated with *P. putida* alone unable to pass on the bacterium to their offspring. Only when we co-inoculated plants with *O. dioscoreae* did we detect *P. putida* in the offspring of plants, albeit at anecdotal frequencies. It is unclear what the mechanisms enforcing specificity of transmission might be, but we speculate that the ability to withstand a quiescent phase of several months before bulbils germinate might be an important factor in transmission success.

This study emphasizes the complexity underlying the regulation of specificity in the *Dioscorea-Orrella* symbiosis. The absence of a strong phenotype of aposymbiotic plants, the permissiveness of the plant for a broad range of bacteria in artificial inoculations, and the fact that specificity seems enforced (at least partially) by microbe–microbe interactions suggest that the barriers to evolving vertically transmitted plant symbioses may be unexpectedly low. This plasticity may explain why leaf symbiosis seems to have evolved independently several times in distinct plant lineages. What adaptations underlie the evolution of vertical transmission in these plant-bacteria associations remain to be discovered.

## Supplementary Material

supplementary_figures_wraf177

Table_S1_Strains_and_Plasmids_wraf177

Table_S2_Oligonucleotides_wraf177

Table_S3_Biolog_wraf177

Table_S4_Leaf_glands_metabolomic_profiles_wraf177

Table_S5_UHPLC-HRMS_annotated_features_wraf177

## Data Availability

All data generated or analyzed during this study are included in this published article and its supplementary information files.

## References

[1] Osman EO, Weinnig AM. Microbiomes and obligate symbiosis of deep-sea animals. *Annu Rev Anim Biosci* 2022;10:151–76. 10.1146/annurev-animal-081621-112021 34843386

[2] Simms EL, Taylor DL, Povich J. et al. An empirical test of partner choice mechanisms in a wild legume–rhizobium interaction. *Proc R Soc B Biol Sci* 2005;273:77–81. 10.1098/rspb.2005.3292 PMC1560009 16519238

[3] Sachs JL, Mueller UG, Wilcox TP. et al. The evolution of cooperation. *Q Rev Biol* 2004;79:135–60. 10.1086/383541 15232949

[4] Nyholm SV, McFall-Ngai MJ. A lasting symbiosis: how the Hawaiian bobtail squid finds and keeps its bioluminescent bacterial partner. *Nat Rev Microbiol* 2021;19:666–79. 10.1038/s41579-021-00567-y 34089010 PMC8440403

[5] Scheuring I, Yu DW. How to assemble a beneficial microbiome in three easy steps. *Ecol Lett* 2012;15:1300–7. 10.1111/j.1461-0248.2012.01853.x 22913725 PMC3507015

[6] Itoh H, Jang S, Takeshita K. et al. Host–symbiont specificity determined by microbe–microbe competition in an insect gut. *Proc Natl Acad Sci* 2019;116:22673–82. 10.1073/pnas.1912397116 31636183 PMC6842582

[7] Worsley SF, Innocent TM, Holmes NA. et al. Competition-based screening helps to secure the evolutionary stability of a defensive microbiome. *BMC Biol* 2021;19:205. 10.1186/s12915-021-01142-w 34526023 PMC8444595

[8] Leeks A, dos Santos M, West SA. Transmission, related, ness, and the evolution of cooperative symbionts. *J Evol Biol* 2019;32:1036–45. 10.1111/jeb.13505 31271473 PMC6852075

[9] Bright M, Bulgheresi S. A complex journey: transmission of microbial symbionts. *Nat Rev Microbiol* 2010;8:218–30. 10.1038/nrmicro2262 20157340 PMC2967712

[10] Pinto-Carbó M, Gademann K, Eberl L. et al. Leaf nodule symbiosis: function and transmission of obligate bacterial endophytes. *Curr Opin Plant Biol* 2018;44:23–31. 10.1016/j.pbi.2018.01.001 29452904

[11] Miller IM . Bacterial leaf nodule symbiosis. *Adv Bot Res* 1990;17:163–234. 10.1016/s0065-2296(08)60134-2 New York Ny, Elsevier.

[12] Danneels B, Blignaut M, Marti G. et al. Cyclitol metabolism is a central feature of *Burkholderia* leaf symbionts. *Environ Microbiol* 2023;25:454–72. 10.1111/1462-2920.16292 36451580

[13] Sieber S, Carlier A, Neuburger M. et al. Isolation and total synthesis of kirkamide, an aminocyclitol from an obligate leaf nodule symbiont. *Angew Chem Int Ed* 2015;54:7968–70. 10.1002/anie.201502696 26033226

[14] Georgiou A, Sieber S, Hsiao C-C. et al. Leaf nodule endosymbiotic *Burkholderia* confer targeted allelopathy to their *Psychotria* hosts. *Sci Rep* 2021;11:22465. 10.1038/s41598-021-01867-2 34789815 PMC8599487

[15] Lemaire B, Vandamme P, Merckx V. et al. Bacterial leaf symbiosis in angiosperms: host specificity without co-speciation. *PLoS One* 2011;6:e24430. 10.1371/journal.pone.0024430 21915326 PMC3168474

[16] Miller IM, Reporter M. Bacterial leaf symbiosis in *Dioscorea sansibarensis*: morphology and ultrastructure of the acuminate leaf glands. *Plant Cell Environ* 1987;10:413–24. 10.1111/1365-3040.ep11603654

[17] Carlier A, Cnockaert M, Fehr L. et al. Draft genome and description of *Orrella dioscoreae* gen. Nov. sp. nov., a new species of *Alcaligenaceae* isolated from leaf acumens of *Dioscorea sansibarensis*. *Syst Appl Microbiol* 2017;40:11–21. 10.1016/j.syapm.2016.10.002 27913074

[18] Acar T, Moreau S, Coen O. et al. Motility-independent vertical transmission of bacteria in leaf symbiosis. *MBio* 2022;13:e0103322. 10.1128/mbio.01033-22 36040028 PMC9600174

[19] Danneels B, Viruel J, McGrath K. et al. Patterns of transmission and horizontal gene transfer in the *Dioscorea sansibarensis* leaf symbiosis revealed by whole-genome sequencing. *Curr Biol* 2021;31:2666–2673.e4. 10.1016/j.cub.2021.03.049 33852872

[20] Acar T, Moreau S, Jardinaud M. et al. The association between *Dioscorea sansibarensis* and *Orrella dioscoreae* as a model for hereditary leaf symbiosis. *PLoS One* 2024;19:e0302377. 10.1371/journal.pone.0302377 38648204 PMC11034651

[21] Debray R, Herbert RA, Jaffe AL. et al. Priority effects in microbiome assembly. *Nat Rev Microbiol* 2022;20:109–21. 10.1038/s41579-021-00604-w 34453137

[22] Pukatzki S, Ma AT, Sturtevant D. et al. Identification of a conserved bacterial protein secretion system in *vibrio cholerae* using the *Dictyostelium* host model system. *Proc Natl Acad Sci USA* 2006;103:1528–33. 10.1073/pnas.0510322103 16432199 PMC1345711

[23] Hood RD, Singh P, Hsu F. et al. A type VI secretion system of *Pseudomonas aeruginosa* targets a toxin to bacteria. *Cell Host Microbe* 2010;7:25–37. 10.1016/j.chom.2009.12.007 20114026 PMC2831478

[24] De Meyer F, Danneels B, Acar T. et al. Adaptations and evolution of a heritable leaf nodule symbiosis between *Dioscorea sansibarensis* and *Orrella dioscoreae*. *ISME J* 2019;13:1831–44. 10.1038/s41396-019-0398-8 30877285 PMC6775992

[25] Boyer F, Fichant G, Berthod J. et al. Dissecting the bacterial type VI secretion system by a genome wide *in silico* analysis: what can be learned from available microbial genomic resources? *BMC Genomics* 2009;10:10. 10.1186/1471-2164-10-104 19284603 PMC2660368

[26] Silverman JM, Agnello DM, Zheng H. et al. Haemolysin coregulated protein is an exported receptor and chaperone of type VI secretion substrates. *Mol Cell* 2013;51:584–93. 10.1016/j.molcel.2013.07.025 23954347 PMC3844553

[27] Pukatzki S, Ma AT, Revel AT. et al. Type VI secretion system translocates a phage tail spike-like protein into target cells where it cross-links actin. *Proc Natl Acad Sci* 2007;104:15508–13. 10.1073/pnas.0706532104 17873062 PMC2000545

[28] Shneider MM, Buth SA, Ho BT. et al. PAAR-repeat proteins sharpen and diversify the type VI secretion system spike. *Nature* 2013;500:350–3. 10.1038/nature12453 23925114 PMC3792578

[29] Zoued A, Durand E, Brunet YR. et al. Priming and polymerization of a bacterial contractile tail structure. *Nature* 2016;531:59–63. 10.1038/nature17182 26909579

[30] Leiman PG, Basler M, Ramagopal UA. et al. Type VI secretion apparatus and phage tail-associated protein complexes share a common evolutionary origin. *Proc Natl Acad Sci* 2009;106:4154–9. 10.1073/pnas.0813360106 19251641 PMC2657435

[31] Basler M, Pilhofer M, Henderson GP. et al. Type VI secretion requires a dynamic contractile phage tail-like structure. *Nature* 2012;483:182–6. 10.1038/nature10846 22367545 PMC3527127

[32] Wang J, Brodmann M, Basler M. Assembly and subcellular localization of bacterial type VI secretion systems. *Ann Rev Microbiol* 2019;73:621–38. 10.1146/annurev-micro-020518-115420 31226022

[33] Colautti J, Kelly SD, Whitney JC. Specialized killing across the domains of life by the type VI secretion systems of *Pseudomonas aeruginosa*. *Biochem J* 2025;482:1–15. 10.1042/BCJ20230240 PMC12133291 39774785

[34] Pietrosiuk A, Lenherr ED, Falk S. et al. Molecular basis for the unique role of the AAA+ chaperone ClpV in type VI protein secretion. *J Biol Chem* 2011;286:30010–21. 10.1074/jbc.M111.253377 21733841 PMC3191042

[35] Cianfanelli FR, Monlezun L, Coulthurst SJ. Aim, load, fire: the type VI secretion system, a bacterial Nanoweapon. *Trends Microbiol* 2016;24:51–62. 10.1016/j.tim.2015.10.005 26549582

[36] Allsopp LP, Bernal P. Killing in the name of: T6SS structure and effector diversity. *Microbiology* 2023;169:001367. 10.1099/mic.0.001367 37490402 PMC10433429

[37] Mougous JD, Gifford CA, Ramsdell TL. et al. Threonine phosphorylation post-translationally regulates protein secretion in *Pseudomonas aeruginosa*. *Nat Cell Biol* 2007;9:797–803. 10.1038/ncb1605 17558395

[38] Miyata ST, Kitaoka M, Brooks TM. et al. *Vibrio cholerae* requires the type VI secretion system virulence factor VasX to kill *Dictyostelium discoideum*. *Infect Immun* 2011;79:2941–9. 10.1128/IAI.01266-10 21555399 PMC3191968

[39] Cheng AT, Ottemann KM, Yildiz FH. *Vibrio cholerae* response regulator VxrB controls colonization and regulates the type VI secretion system. *PLoS Pathog* 2015;11:e1004933. 10.1371/journal.ppat.1004933 26000450 PMC4441509

[40] Clark DJ, Maaløe O. DNA replication and the division cycle in *Escherichia coli*. *J Mol Biol* 1967;23:99–112. 10.1016/S0022-2836(67)80070-6

[41] Heuer H, Krsek M, Baker P. et al. Analysis of actinomycete communities by specific amplification of genes encoding 16S rRNA and gel-electrophoretic separation in denaturing gradients. *Appl Environ Microbiol* 1997;63:3233–41. 10.1128/aem.63.8.3233-3241.1997 9251210 PMC168621

[42] Sana TG, Notopoulou A, Puygrenier L. et al. Structures and roles of BcsD and partner scaffold proteins in proteobacterial cellulose secretion. *Curr Biol* 2024;34:106–116.e6. 10.1016/j.cub.2023.11.057 38141614

[43] Volke DC, Wirth NT, Nikel P. Rapid genome engineering of *pseudomonas* assisted by fluorescent markers and tractable curing of plasmids. *Bio-Protoc J* 2021;11:11. 10.21769/BioProtoc.3917 PMC7952922 33732804

[44] Carlier A, Moreau S. CarlierLab/NanoSeq: NanoSeq v0.1.3. *Zenodo* 2024. 10.5281/zenodo.13641971

[45] Vaas LAI, Sikorski J, Hofner B. et al. Opm: an R package for analysing OmniLog® phenotype microarray data. *Bioinformatics* 2013;29:1823–4. 10.1093/bioinformatics/btt291 23740744

[46] Sprouffske K, Wagner A. Growthcurver: an R package for obtaining interpretable metrics from microbial growth curves. *BMC Bioinformatics* 2016;17:172. 10.1186/s12859-016-1016-7 27094401 PMC4837600

[47] R Core Team. R . A Language and Environment for Statistical Computing. Vienna, Austria: R Foundation for Statistical Computing, 2024, 2024.

[48] Tsugawa H, Cajka T, Kind T. et al. MS-DIAL: data-independent MS/MS deconvolution for comprehensive metabolome analysis. *Nat Methods* 2015;12:523–6. 10.1038/nmeth.3393 25938372 PMC4449330

[49] Fraisier-Vannier O, Chervin J, Cabanac G. et al. MS-CleanR: a feature-filtering workflow for untargeted LC–MS based metabolomics. *Anal Chem* 2020;92:9971–81. 10.1021/acs.analchem.0c01594 32589017

[50] Murray K, Duggleby CJ, Williams P. et al. The metabolism of benzoate and methylbenzoates via the meta-cleavage pathway by pseudomonas arvilla mt-2. *Eur J Biochem* 1972;28:301–10. 10.1111/j.1432-1033.1972.tb01914.x 4342906

[51] Bagdasarian M, Lurz R, Rückert B. et al. Specific-purpose plasmid cloning vectors II. Broad host range, high copy number, RSF 1010-derived vectors, and a host-vector system for gene cloning in pseudomonas. *Gene* 1981;16:237–47. 10.1016/0378-1119(81)90080-9 6282695

[52] Nogales J, Mueller J, Gudmundsson S. et al. High-quality genome-scale metabolic modelling of pseudomonas putida highlights its broad metabolic capabilities. *Environ Microbiol* 2020;22:255–69. 10.1111/1462-2920.14843 31657101 PMC7078882

[53] Nogales J, Palsson BØ, Thiele I. A genome-scale metabolic reconstruction of pseudomonas putida KT2440: i JN746 as a cell factory. *BMC Syst Biol* 2008;2:79. 10.1186/1752-0509-2-79 18793442 PMC2569920

[54] Puchałka J, Oberhardt MA, Godinho M. et al. Genome-scale reconstruction and analysis of the pseudomonas putida KT2440 metabolic network facilitates applications in biotechnology. *PLoS Comput Biol* 2008;4:e1000210. 10.1371/journal.pcbi.1000210 18974823 PMC2563689

[55] Nikel PI, Chavarría M, Fuhrer T. et al. Pseudomonas putida KT2440 strain metabolizes glucose through a cycle formed by enzymes of the Entner-Doudoroff, Embden-Meyerhof-Parnas, and pentose phosphate pathways. *J Biol Chem* 2015;290:25920–32. 10.1074/jbc.M115.687749 26350459 PMC4646247

[56] Sohn SB, Kim TY, Park JM. et al. In silico genome-scale metabolic analysis of pseudomonas putida KT2440 for polyhydroxyalkanoate synthesis, degradation of aromatics and anaerobic survival. *Biotechnol J* 2010;5:739–50. 10.1002/biot.201000124 20540110

[57] Su Y, Lin H-C, Teh LS. et al. Rational engineering of a synthetic insect-bacterial mutualism. *Curr Biol* 2022;32:3925–3938.e6. 10.1016/j.cub.2022.07.036 35963240 PMC10080585

[58] Koga R, Moriyama M, Onodera-Tanifuji N. et al. Single mutation makes Escherichia coli an insect mutualist. *Nat Microbiol* 2022;7:1141–50. 10.1038/s41564-022-01179-9 35927448 PMC9352592

[59] Expert D . Withholding and exchanging iron: interactions between Erwinia spp. and their plant hosts. *Annu Rev Phytopathol* 1999;37:307–34. 10.1146/annurev.phyto.37.1.307 11701826

[60] Carlier A, Fehr L, Pinto-Carbó M. et al. The genome analysis of Candidatus Burkholderia crenata reveals that secondary metabolism may be a key function of the Ardisia crenata leaf nodule symbiosis. *Environ Microbiol* 2016;18:2507–22. 10.1111/1462-2920.13184 26663534

[61] Molina-Santiago C, Udaondo Z, Cordero BF. et al. Interspecies cross-talk between co-cultured pseudomonas putida and Escherichia coli. *Environ Microbiol Rep* 2017;9:441–8. 10.1111/1758-2229.12553 28585781

[62] Dutta S, Lee YH. High-throughput identification of genes influencing the competitive ability to obtain nutrients and performance of biocontrol in pseudomonas putida JBC17. *Sci Rep* 2022;12:872. 10.1038/s41598-022-04858-z 35042886 PMC8766522

[63] Jurėnas D, Journet L. Activity, deli, very, and diversity of type VI secretion effectors. *Mol Microbiol* 2020;115:383–94. 10.1111/mmi.14648 33217073

[64] Bernal P, Allsopp LP, Filloux A. et al. The pseudomonas putida T6SS is a plant warden against phytopathogens. *ISME J* 2017;11:972–87. 10.1038/ismej.2016.169 28045455 PMC5363822

[65] Chen W-J, Kuo T-Y, Hsieh F-C. et al. Involvement of type VI secretion system in secretion of iron chelator pyoverdine in Pseudomonas taiwanensis. *Sci Rep* 2016;6:32950. 10.1038/srep32950 27605490 PMC5015096

[66] Worrall LJ, Majewski DD, Strynadka NCJ. Structural insights into type III secretion systems of the bacterial flagellum and injectisome. *Ann Rev Microbiol* 2023;77:669–98. 10.1146/annurev-micro-032521-025503 37713458

[67] Songwattana P, Chaintreuil C, Wongdee J. et al. Identification of type III effectors modulating the symbiotic properties of Bradyrhizobium vignae strain ORS3257 with various Vigna species. *Sci Rep* 2021;11:4874. 10.1038/s41598-021-84205-w 33649428 PMC7921652

[68] Leigh EG Jr . The evolution of mutualism. *J Evol Biol* 2010;23:2507–28. 10.1111/j.1420-9101.2010.02114.x 20942825

[69] Wilson DS . Biological communities as functionally organized units. *Ecology* 1997;78:2018–24. 10.1890/0012-9658(1997)078[2018:BCAFOU]2.0.CO;2

