# Supplementary material for: Artificial symbiont replacement in a vertically transmitted plant symbiosis reveals a role for microbe–microbe interactions in enforcing specificity
Source: ISME J. 2025 Aug 19;19(1):wraf177. doi: 10.1093/ismejo/wraf177 (PMC12411853; doi:10.1093/ismejo/wraf177)
Supplement: supplementary_figures_wraf177 [file supplementary_figures_wraf177.pdf]

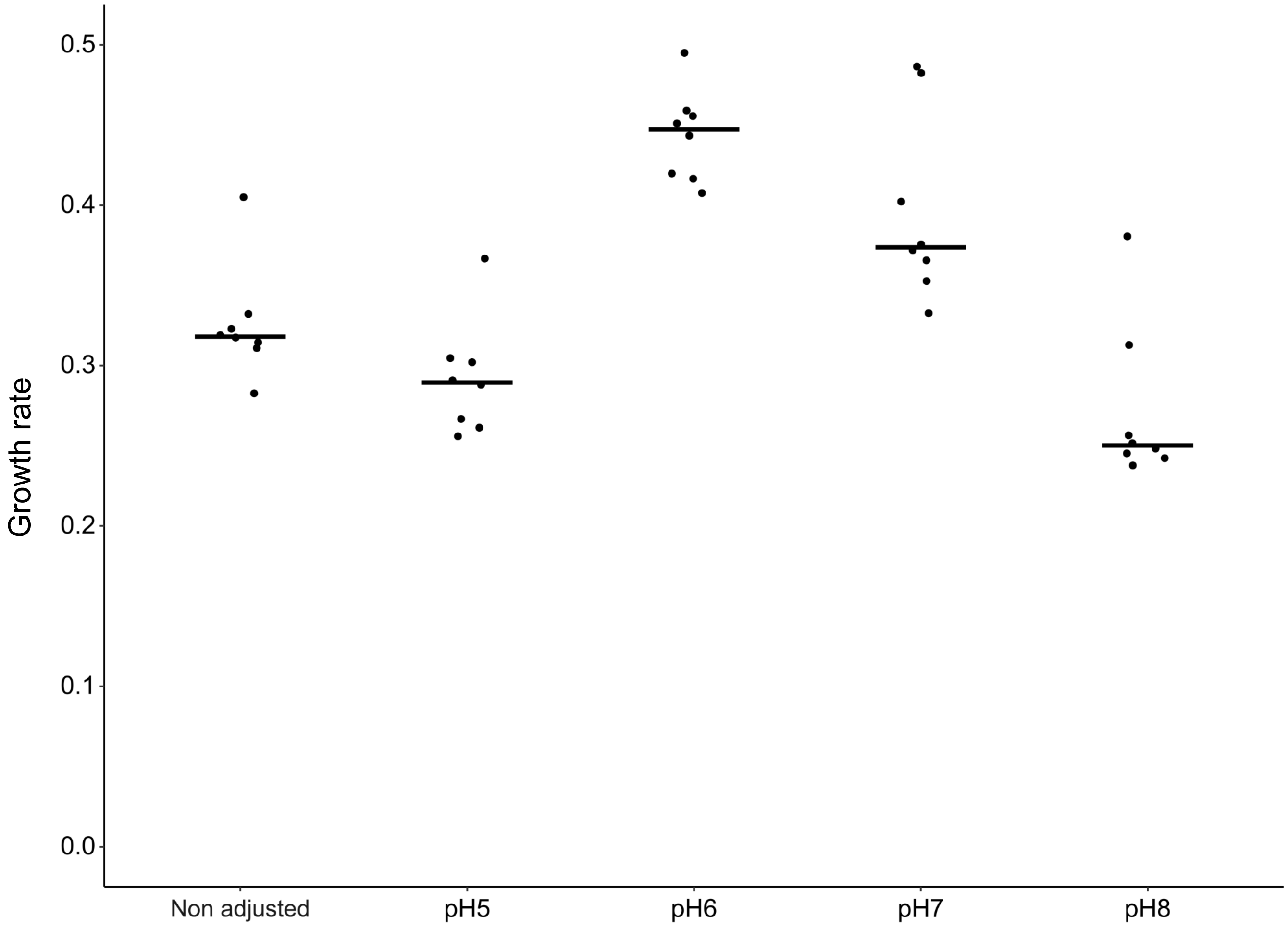

Figure S1. Growth of *O. dioscoreae* at various pH. Growth of *O. dioscoreae* R-71412 was monitored for 48 hours in LB medium supplemented with 10 mM trisodium citrate from pH5 to pH8. Medium was buffered with MES, MOPS or EPPS buffer as appropriate for the target pH; non adjusted medium was used as a control. Growth rate was calculated using R package Growthcurver (Sprouffske et Wagner 2016). Horizontal lines represent the median.

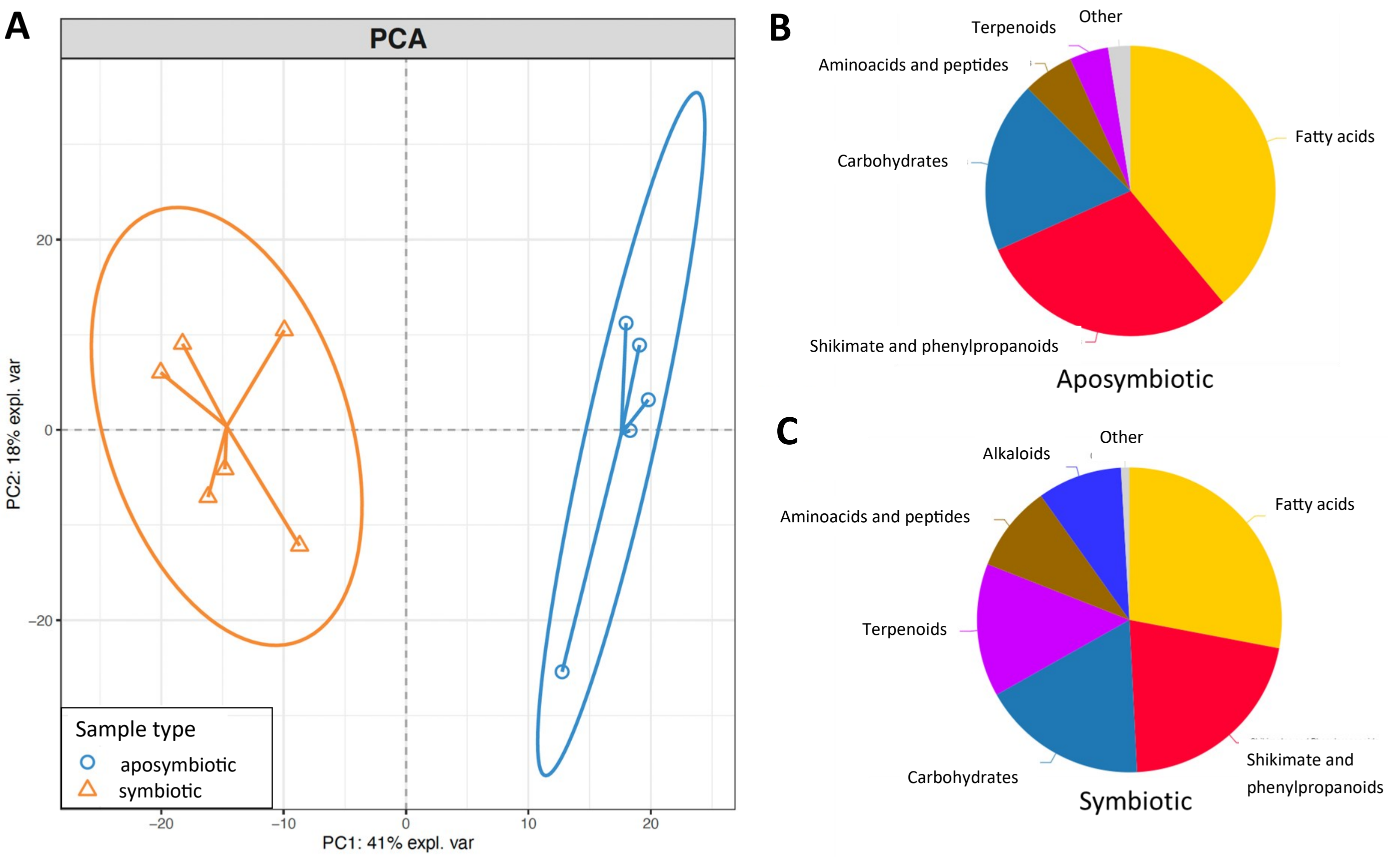

Figure S2. Metabolic profile analysis of symbiotic and aposymbiotic *D. sansibarensis* leaf glands. A. Principal component analysis of metabolomic profiles based on peak intensity for each detected feature (Table S4). B. Distribution of the major metabolite classes (PathwayNP) as classified by NPclassifier (Kim et al. 2021), from mean peak area of annotated features in each group in aposymbiotic leaf gland extracts and C. in symbiotic leaf gland extracts. Only features that are differentially abundant (FDR < 0.05) are considered in panels B and C.

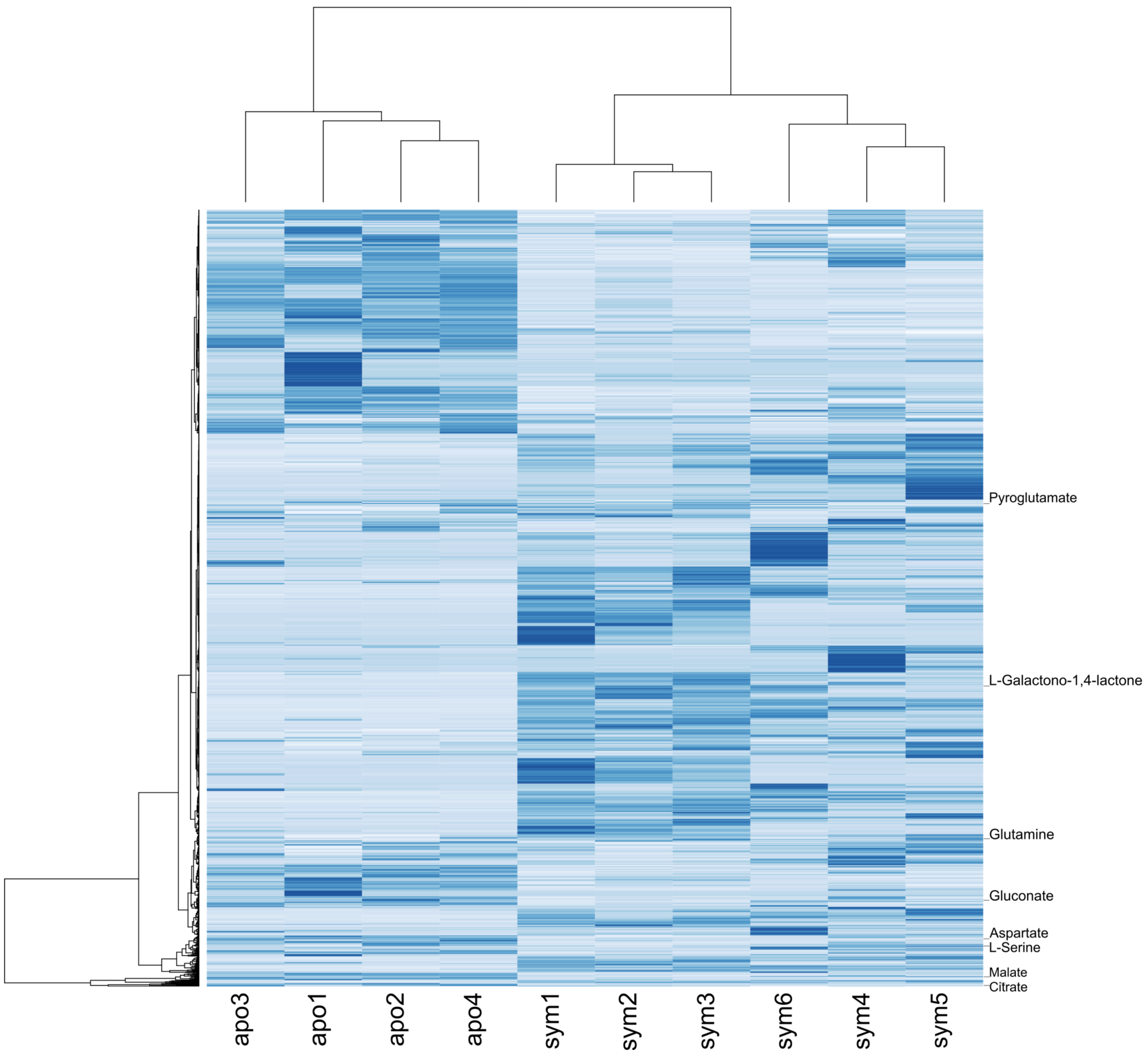

Figure S3. Heatmap of features detected by HPLC MS/MS of symbiotic and aposymbiotic *Dioscorea sansibarensis* leaf glands. Each row represents a unique LC-MS/MS feature, and source peak intensity data is available in Table S4. Labels on the right-hand side indicate potential carbon on nitrogen sources utilized by *O. dioscoreae* as listed in Table 2. Darker colors indicate higher peak intensities. Clustering and graphical representation were done using the « heatmap » function of R v.4.4.1, with normalization per row based on Z-score.

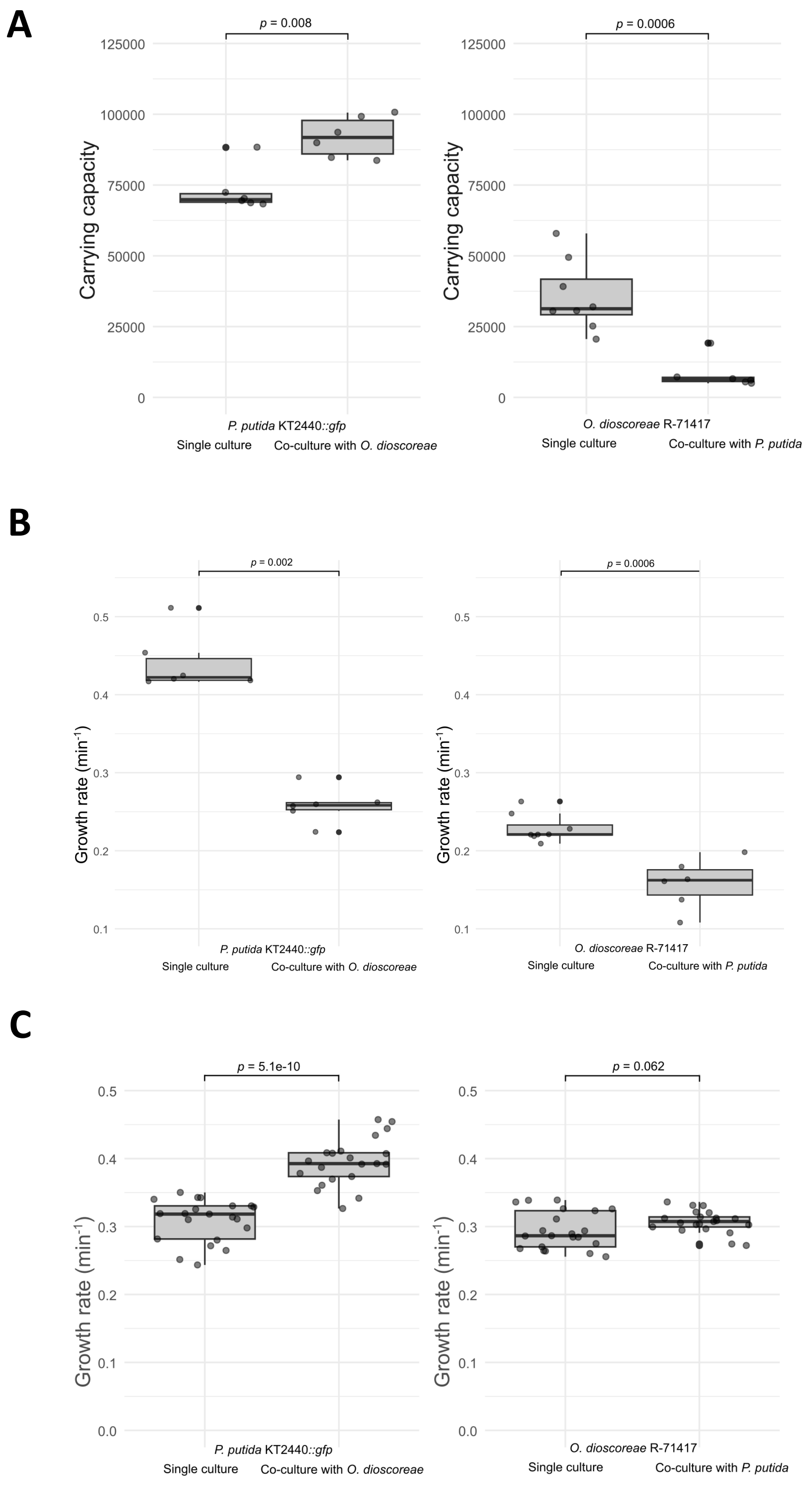

Figure S4. Growth characteristics of *O. dioscoreae* R-71417 and *P. putida* K2440*::gfp* in single and co-culture in LB and ABCY media. LB or ABCY cultures in microtiter plates were inoculated with strains *P. putida* KT2440*::gfp* and mCherrry–tagged *O. dioscoreae* R-71417 together or separately. GFP– and mCherry-specific fluorescence were monitored for 25h. Relative fluorescence values were analyzed with the Growthcurver R package to derive growth parameters *K* (carrying capacity) and *r* (growth rate) for each culture (Sprouffske et Wagner 2016). A. Carrying capacity of *P. putida* KT2440*::gfp* and *O. dioscoreae* R-71417 in single or co-cultures in ABCY medium. B. Growth rate of *P. putida* KT2440*::gfp* and *O. dioscoreae* R-71417 in single or co-cultures in ABCY medium. C. Growth rate of *P. putida* KT2440*::gfp* and *O. dioscoreae* R-71417 in single or co-cultures in LB medium. Horizontal bars indicate *P* values (Wilcoxon rank sum test).

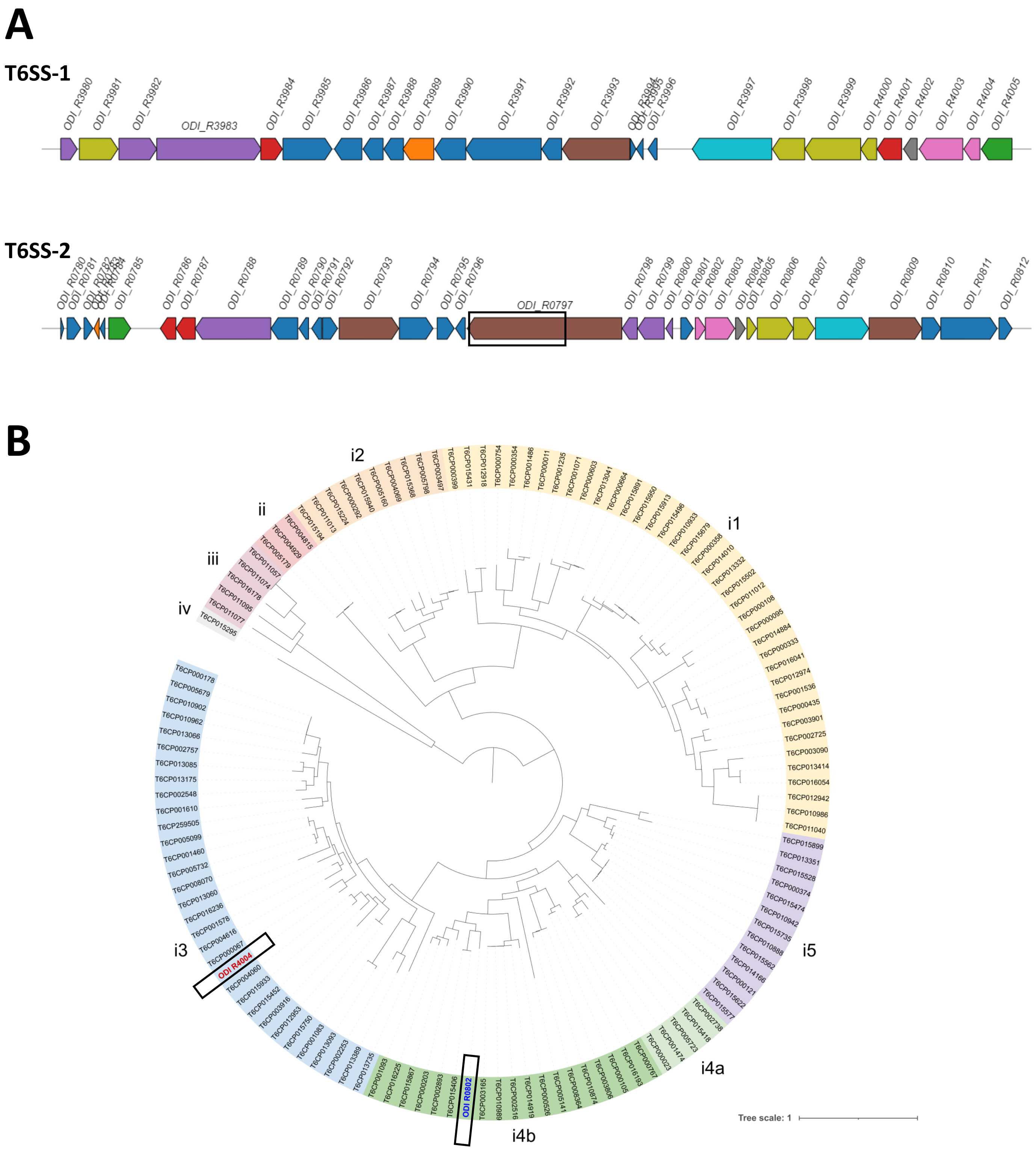

Figure S5. Type VI Secretion System of *O. dioscoreae*. A. Genetic structure of the two complete T6SS gene clusters of *O. dioscoreae* LMG29303^{T}: T6SS-1 (locus_tag ODI_R3980 to ODI_R4005) and T6SS-2 (locus tag ODI_0780 to ODI_R0812). Putative protein functions and domains are color-coded: baseplate proteins in green, integral membrane complex proteins in purple, contractile sheath in pink, Hcp in grey, VgrG in brown, PAAR in orange, ClpV AAA+ ATPase in yellow, accessory proteins in red and unknown functions in blue. The VgrG-domain protein encoded by gene ODI_R0797 possesses a partial eukaryotic Med15 domain in C-terminal (black box). Gene cluster annotated sequences were downloaded from NCBI, and processed with R package GeneViewer (https://github.com/nvelden/geneviewer). B. Phylogenetic classification of TssB sequences. Experimentally verified TssB amino-acid sequences were downloaded from the SecReT6 database (Zhang et al. 2023) and aligned using MAFFT v7.475. A phylogenetic tree was constructed with FastTree v2.1.11 with default settings, and the tree was displayed in iTol (Letunic and Bork, 2021). Colored ranges indicate T6SS types, and proteins *O. dioscoreae* proteins ODI_R4004 (T6SS-1) and ODI_R0802 (T6SS-2) are displayed in red and blue fonts, respectively.

References:

Letunic I, Bork P. Interactive Tree Of Life (iTOL) v5: an online tool for phylogenetic tree display and annotation. Nucleic Acids Res. 2021 Jul 2;49(W1):W293-W296.

Zhang J, Guan J, Wang M, Li G, Djordjevic M, Tai C, Wang H, Deng Z, Chen Z, Ou HY. SecReT6 update: a comprehensive resource of bacterial Type VI Secretion Systems. Sci China Life Sci. 2023 Mar;66(3):626-634.

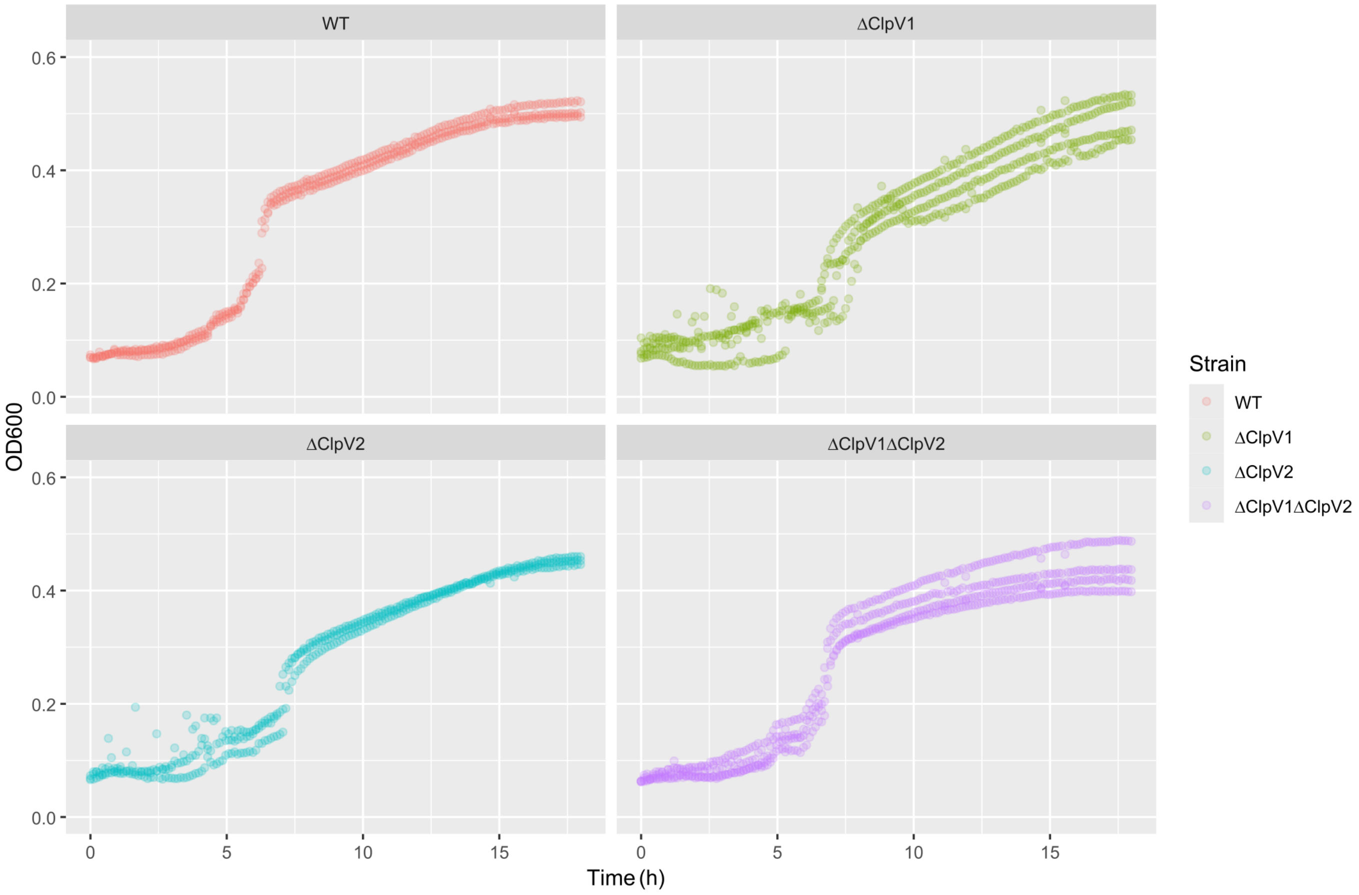

Figure S6. Growth of *O. dioscoreae* R-71412 and derivatives. Cultures in TSB medium were inoculated with *O. dioscoreae* strains R-71412 (WT), Δ3997 (*ΔclpV1*), Δ0808 (*ΔclpV2*) and Δ3997Δ0808 (*ΔclpV1ΔclpV2*). Growth was monitored in microtiter plates filled with 200 µL of culture at 28°C with shaking. Absorbance at 600nm was measured automatically every 6 min. The experiment was performed with 3 or 4 replicates per strain. Growth characteristics do not differ significantly between the strains (pairwise wilcoxon rank sum test computed on areas under the curves for each sample, with Benjamini-Hochberg correction for multiple testing, $p > 0.05$).

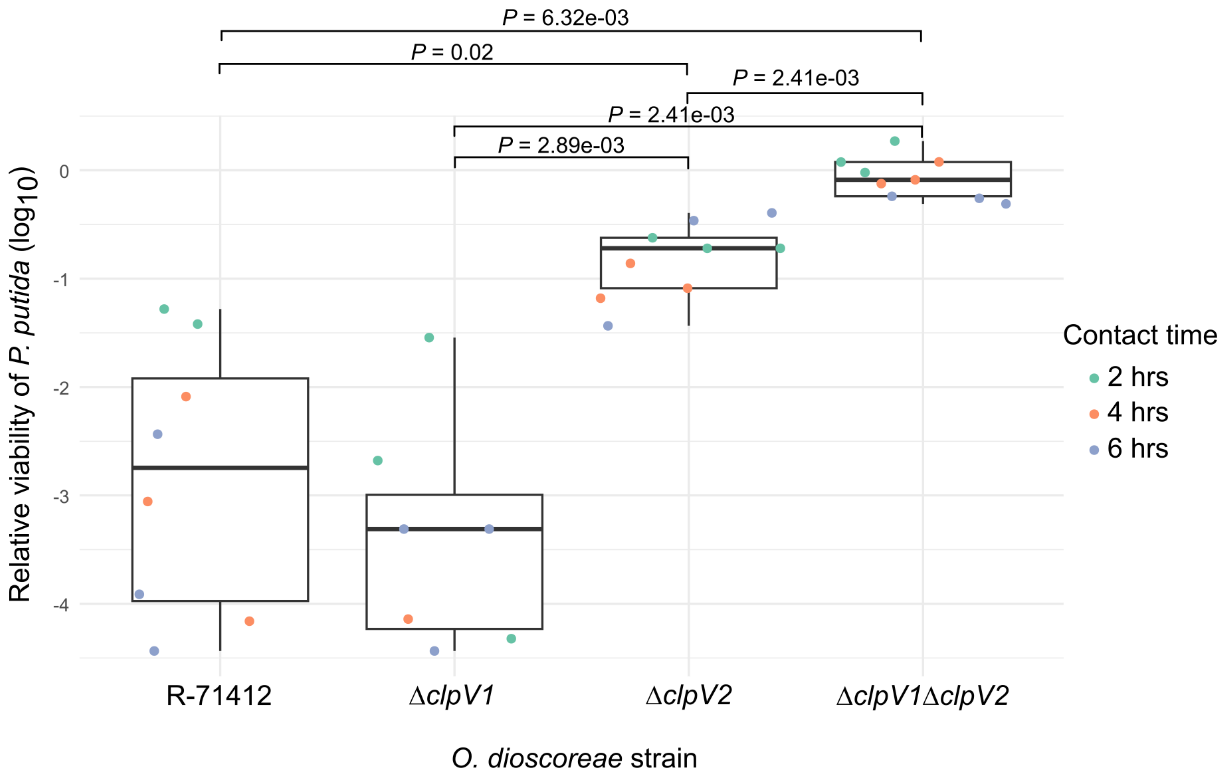

Figure S7. Contact-dependent killing of *P. putida* KT2440*::gfp* by *O. dioscoreae*. Cultures were prepared as described in the Materials and Methods section. Briefly, bacteria were washed once and resuspended in a 0.4% Nacl solution adjusted to OD_{600nm} = 10 for *P. putida* and OD_{600nm} = 50 for *O. dioscoreae*. *P. putida* cells were then mixed with a volume of 0.4% NaCl or a suspension of *O. dioscoreae* to yield a final ratio of 1:5 (w/w, *P. putida* : *O. dioscoreae*). Suspensions were spotted on pre-warmed TSA plates and inculated at 28°C for 2, 4 or 6 hours. Bacteria were retrieved and cfu counts were estimated by serial dilution on selective media. Relative viability ratios were calculated as the cfu counts of *P. putida* KT2440*::gfp* for each treatment, divided by the cfu counts of *P. putida* KT2440*::gfp* in the control without *O. dioscoreae*. Bars indicate *P* values according to a pairwise Yuenn's test for trimmed means with Benjamini Hochberg correction for multiple testing. Only *P* values adjusted < 0.05 are shown.

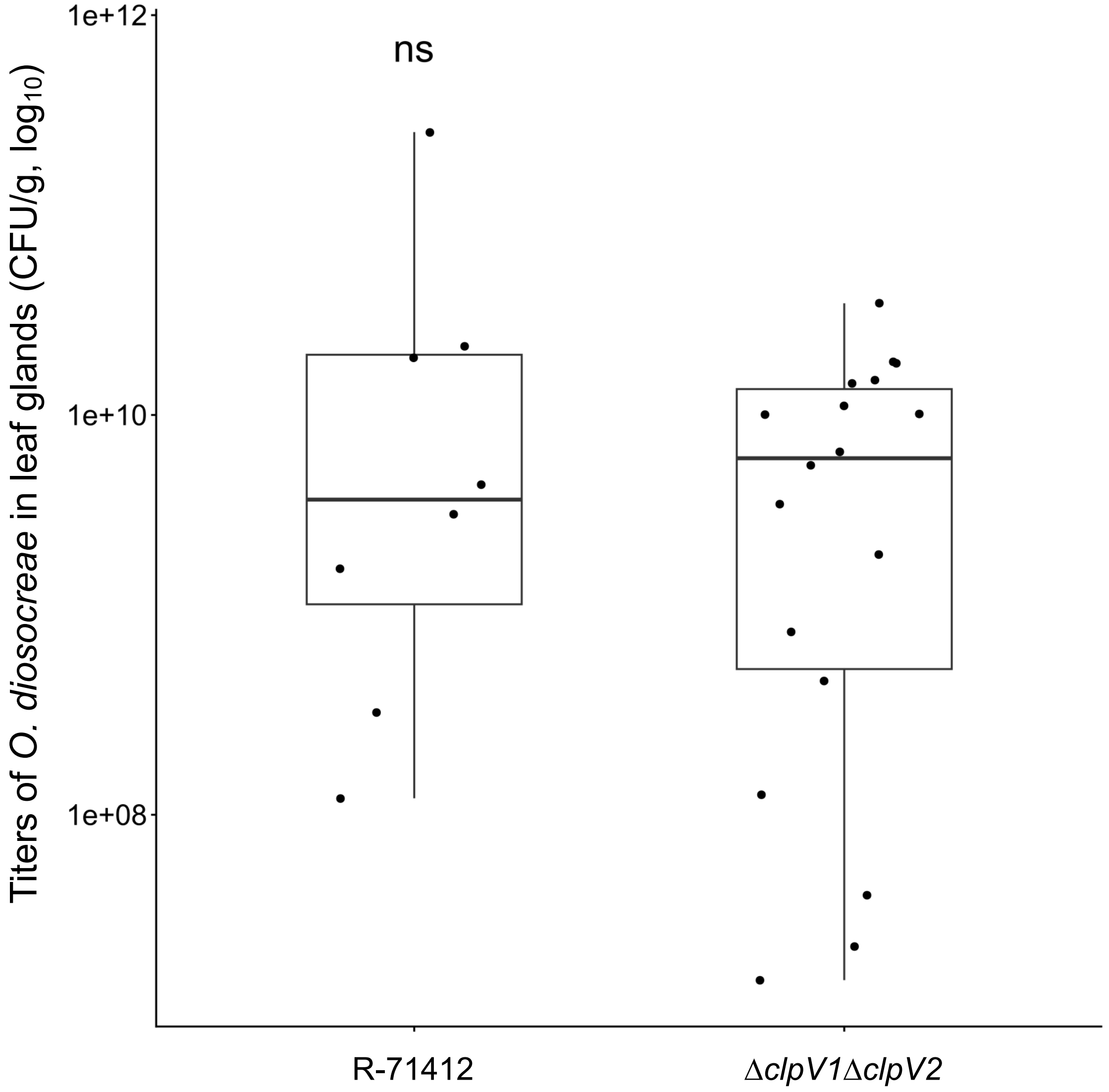

Figure S8. *D. sansibarensis* leaf glands colonization by *O. dioscoreae* strains. Aposymbiotic *D. sansibarensis* were inoculated with *O. dioscoreae* strains R-71412, the T6SS-mutant *ΔclpV1ΔclpV2*. Newly grown leaf glands were weighed and macerated. Serial dilutions were plated on selective media to quantify bacterial load. Statistical significance calculated with Student's t-test (significance levels: ns p-value >0.05; * p-value <=0.05; ** p-value <=0.01; *** p-value <=0.001; **** p-value <=0.0001).

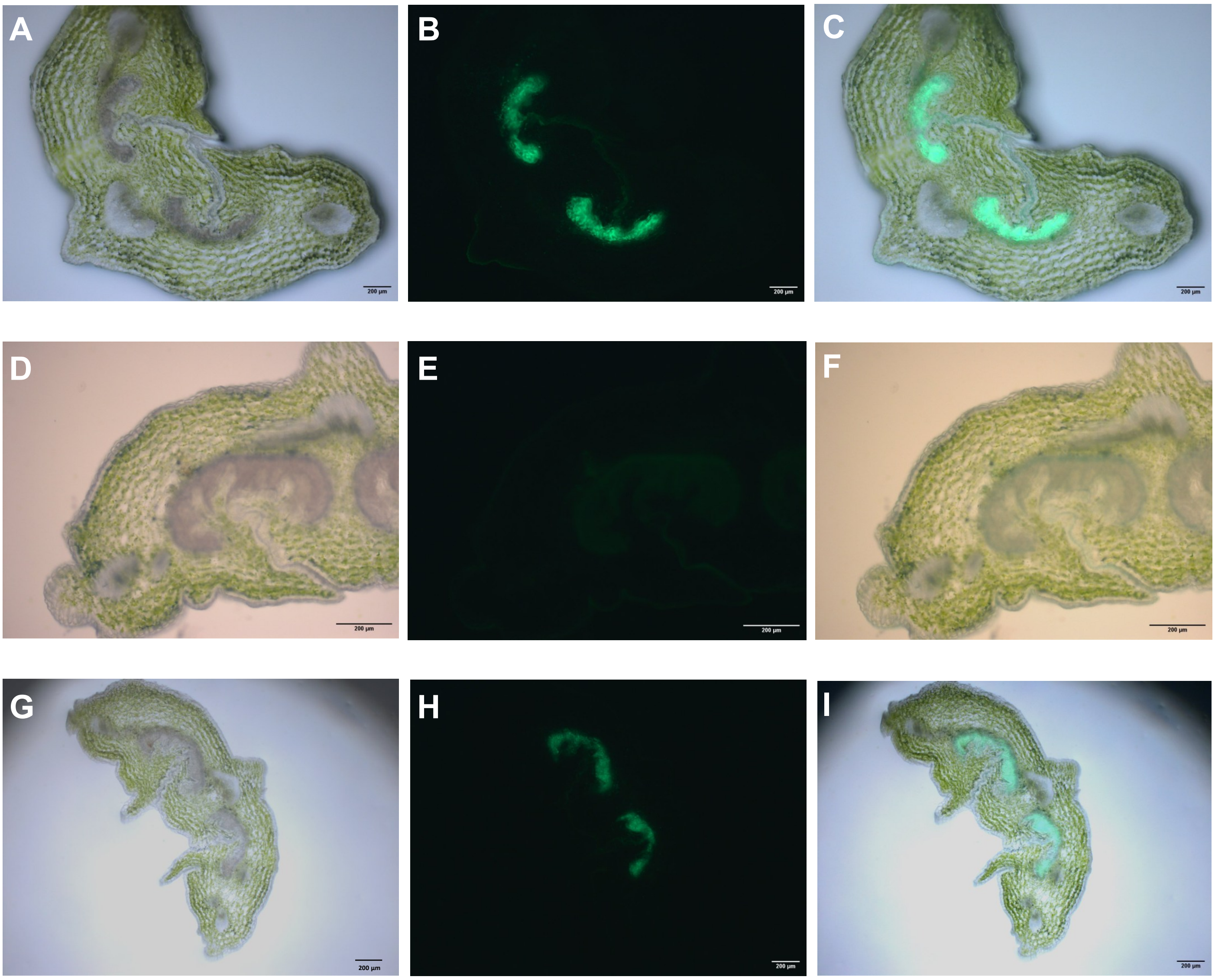

Figure S9. Colonization of *D. sansibarensis* by *P. putida* KT2440*::gfp* in single or co-inoculation with *O. dioscoreae*. A. Cross-section of a *D. sansibarensis* acumen colonized by *P. putida* KT2440*::gfp* in bright field microscopy. B. The same sample under epifluorescence. C. Merged image of A and B. D. Cross-section of a *D. sansibarensis* acumen colonized by *P. putida* KT2440*::gfp* and *O. dioscoreae* R-71412 in bright field microscopy. E. The same sample under epifluorescence. F. Merged image of D and E. G. Cross-section of a *D. sansibarensis* acumen colonized by *P. putida* KT2440*::gfp* and *O. dioscoreae ΔclpV1ΔclpV2* in bright field microscopy. H. The same sample under epifluorescence. I. Merged image of G and H.

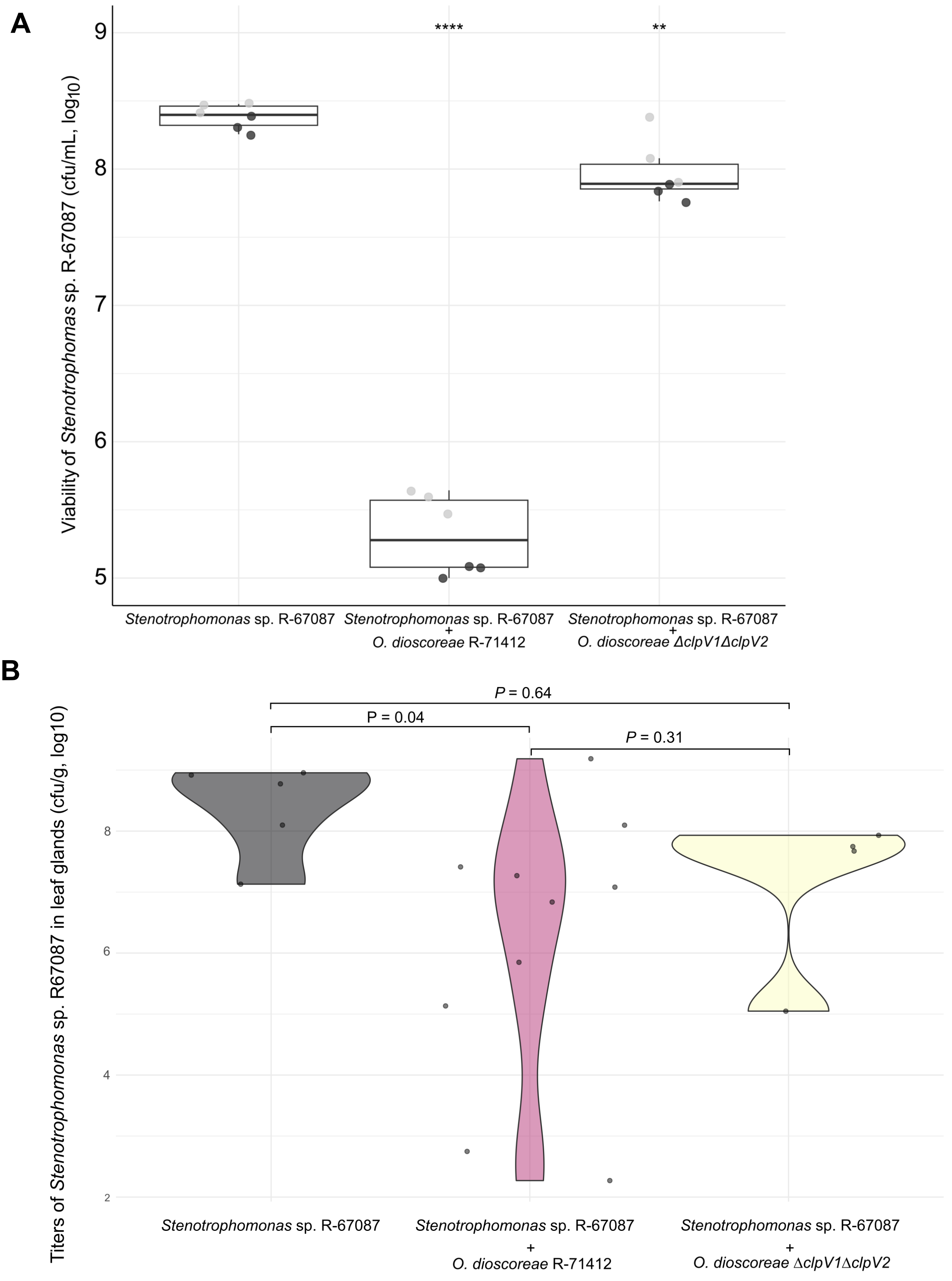

Figure S10. Contact-dependent competition assays of *O. dioscoreae* against *Stenotrophomonas sp.* R-67087. Survival of *Stenotrophomonas sp.* R-67087 after 4 hours of co-culture on TSA medium in the presence of *O. dioscoreae* R-71412 and T6SS-impaired mutant *ΔclpV1ΔclpV2* as measured by cfu counting. Colors of the points indicate the replicate. Significance from Student's T-test with the R-67087 alone as group of reference (significance levels: ns p-value>0.05; * p-value<=0.05; ** p-value<=0.01; ***p-value<=0.001; **** p-value<=0.0001. B. Colonization (log10 cfu/g) of *D. sansibarensis* acumens after inoculation of plants with *Stenotrophomonas* sp. R-67087 in single inoculation or co-inoculation with *O. dioscoreae* R-71412 (wild-type) or *O. dioscoreae ΔclpV1ΔclpV2*. In this experiment, plants were inoculated successively with cell suspensions of *O. dioscoreae* and *S.* sp. R-67087. Statistical significance of colonization variation was calculated with a Student's T-test. Bars indicate *P* values.
