# Supplementary material for: Artificial symbiont replacement in a vertically transmitted plant symbiosis reveals a role for microbe–microbe interactions in enforcing specificity
Source: ISME J. 2025 Aug 19;19(1):wraf177. doi: 10.1093/ismejo/wraf177 (PMC12411853; doi:10.1093/ismejo/wraf177)
Supplement: Table_S1_Strains_and_Plasmids_wraf177 [file table_s1_strains_and_plasmids_wraf177.docx]

**Table S1. List of strains and plasmids used in this study.**

| **Strain** | **Description** | **Growth conditions** | **Reference or source** |
| --- | --- | --- | --- |
| Top10 | *ΔlacX74 araΔ139 Δ(ara-leu)* | LB, 37°C | Thermo Fisher Scientific |
| DB3.1 *λpir* | Host strain for pSNW2 and derivatives. *F^-^gyrA462 endA1 glnV44 Δ(sr1-recA) mcrB mrr hsdS20 (r_B_^-^m_B_^-^) ara14 galK2 lacY1 proA2 rpsL20 xyl5 Δleu mtl1 pir+* | LB, 37°C | Thermo Fisher Scientific |
| R-71412 | *Orrella dioscoreae.* Spontaneous nalidixic acid resistant strain derived from type strain LMG 29303^T^. | TSA + Nalidixic acid 30 µg/mL, aerobic, 28°C | (De Meyer et al. 2019) |
| R-71417 | *Orrella dioscoreae.* mCherry-tagged derivative of R-71412 | TSA + Nalidixic acid 30 µg/ml + Gentamicin 20 µg/mL, aerobic, 28°C | (Acar et al. 2022) |
| ∆3997 | *Orrella dioscoreae.* Deletion mutant in *clpV1* gene, derivative of strain R-71412 | TSA + Nalidixic acid 30 µg/mL, aerobic, 28°C | This study |
| ∆0808 | *Orrella dioscoreae*. Deletion mutant in *clpV2*gene, derivative of R-71412 | TSA + Nalidixic acid 30 µg/mL, aerobic, 28°C | This study |
| ∆3997∆0808 | *Orrella dioscoreae.* Deletion mutant in *clpV1* and *clpV2* genes, derivative of R-71412 | TSA + Nalidixic acid 30 µg/mL, aerobic, 28°C | This study |
| ∆3997/*clpV1+* | *Orrella dioscoreae* strain ∆3997 harboring pSEVA2313_R3997 | TSA + Nalidixic acid 30 µg/mL + Kanamycin 50 µg/mL, aerobic, 28°C | This study |
| ∆0808/*clpV2+* | *Orrella dioscoreae* strain ∆0808 harboring pSEVA2313_R0808 | TSA + Nalidixic acid 30 µg/mL + Kanamycin 50 µg/mL, aerobic, 28°C | This study |
| ∆3997∆0808/*clpV1+* | *Orrella dioscoreae* strain ∆3997∆0808 harboring pSEVA2313_R3997 | TSA + Nalidixic acid 30 µg/mL + Kanamycin 50 µg/mL, aerobic, 28°C | This study |
| ∆3997∆0808/*clpV2+* | *Orrella dioscoreae* strain ∆3997∆0808 harboring pSEVA2313_R0808 | TSA + Nalidixic acid 30 µg/mL + Kanamycin 50µg/mL, aerobic, 28°C | This study |
| KT2440::*gfp* | *Pseudomonas putida.* P_14g_(BCD2)->msfGFP (monomeric superfolder GFP under control of a P14g promoter and a biscistronic design), chromosomally integrated into a landing pad between genes PP_0013 and PP_5421 | LB, 28°C | Gift from Nicolas Krink (Nikel lab DTU) |
| **Plasmid** |  |  |  |
| pQURE6 | Conditionally replicating broad host range plasmid encoding the homing endonuclease I-SceI. Used for counter-selection of merodiploid clones. | LB + Gentamicin 20 µg/mL, 37°C | (Volke, Wirth, and Nikel 2021) |
| pSEVA2313 | Broad host range vector, *Km^R^* | LB + Kanamycin 25 µg/mL, 37°C | (Silva-Rocha et al. 2013) |
| pSNW2 | Suicide vector, *R6K ori, Km^R^.* Contains I-SceI restriction sites. | LB + Kanamycin 25 µg/mL, 37°C | (Volke, Wirth, and Nikel 2021) |
| pUX-BF13 | R6K replicon -based helper plasmid providing the Tn7 transposition function in trans. *Ap^r^, mob^+^* | LB + Ampicillin 100 µg/mL, 37°C | (Choi and Schweizer 2005) |
| pSNW2_mut_R0808 | 500 bp fragments flanking *O. dioscoreae* gene ODI_R0808 cloned into pSNW2. | LB + Kanamycin 25 µg/mL, 37°C | This study |
| pSNW2_mut_R3997 | 500 bp fragments flanking *O. dioscoreae* gene ODI_R3997 cloned into pSNW2. | LB + Kanamycin 25 µg/mL, 37°C | This study |
| pSEVA2313_R0808 | *O. dioscoreae* ODI_R0808 gene cloned into broad host range vector pSEVA2313. | LB + Kanamycin 25µg/mL, 37°C | This study |
| pSEVA2313_R3997 | *O. dioscoreae* ODI_R3997 gene cloned into broad host range vector pSEVA2313. | LB + Kanamycin 25µg/mL, 37°C | This study |

REFERENCES

Acar, Tessa, Sandra Moreau, Olivier Coen, Frédéric De Meyer, Olivier Leroux, Marine Beaumel, Paul Wilkin, and Aurélien Carlier. 2022. “Motility-Independent Vertical Transmission of Bacteria in Leaf Symbiosis.” *mBio* 13 (5): e0103322. https://doi.org/10.1128/mbio.01033-22.

Choi, Kyoung-Hee, and Herbert P. Schweizer. 2005. “An Improved Method for Rapid Generation of Unmarked *Pseudomonas Aeruginosa* Deletion Mutants.” *BMC Microbiology* 5 (May):30. https://doi.org/10.1186/1471-2180-5-30.

De Meyer, Frédéric, Bram Danneels, Tessa Acar, Rado Rasolomampianina, Mamy Tiana Rajaonah, Vololoniaina Jeannoda, and Aurélien Carlier. 2019. “Adaptations and Evolution of a Heritable Leaf Nodule Symbiosis between *Dioscorea Sansibarensis* and *Orrella Dioscoreae*.” *The ISME Journal* 13 (7): 1831–44. https://doi.org/10.1038/s41396-019-0398-8.

Silva-Rocha, Rafael, Esteban Martínez-García, Belén Calles, Max Chavarría, Alejandro Arce-Rodríguez, Aitor de las Heras, A. David Páez-Espino, et al. 2013. “The Standard European Vector Architecture (SEVA): A Coherent Platform for the Analysis and Deployment of Complex Prokaryotic Phenotypes.” *Nucleic Acids Research* 41 (D1): D666–75. https://doi.org/10.1093/nar/gks1119.

Volke, Daniel C., Nicolas T. Wirth, and Pablo Nikel. 2021. “Rapid Genome Engineering of *Pseudomonas* Assisted by Fluorescent Markers and Tractable Curing of Plasmids.” *BIO-PROTOCOL* 11 (4). https://doi.org/10.21769/BioProtoc.3917.
