# Supplementary material for: Artificial symbiont replacement in a vertically transmitted plant symbiosis reveals a role for microbe–microbe interactions in enforcing specificity
Source: ISME J. 2025 Aug 19;19(1):wraf177. doi: 10.1093/ismejo/wraf177 (PMC12411853; doi:10.1093/ismejo/wraf177)
Supplement: Table_S2_Oligonucleotides_wraf177 [file table_s2_oligonucleotides_wraf177.docx]

**Table S2. List of oligonucleotides used in this study.**

| Primer | Sequence | Used for |
| --- | --- | --- |
| **27F** | AGAGTTTGATCCTGGCTCAG | Taxonomic identification by 16S rRNA sequencing |
| **1492R** | TACGGYTACCTTGTTACGACTT |  |
| **R3997_L_fwd** | TGAATTCGAGCTCGGTACCCGCGGGCTCGAGCAGCTGGGC | PCR amplification of flanking sequences and construction of *O. dioscoreae* ODI_R3997 deletion mutant. |
| **R3997_L_rev** | TCACAGAGCCCAGCGCCATGTGACACGGGAGCCACGCCAT |  |
| **R3997_R_fwd** | ATGGCGTGGCTCCCGTGTCACATGGCGCTGGGCTCTGTGA |  |
| **R3997_R_rev** | GTCGACTCTAGAGGATCCCCTGCGCATCCAGGAGTTCGTG |  |
| **R0808_L_fwd** | TGAATTCGAGCTCGGTACCCGCGCGTGCTGTCCGACTACT | PCR amplification of flanking sequences and construction of *O. dioscoreae* ODI_R0808 deletion mutant. |
| **R0808_L_rev** | GCTGGTGCTCCCTCAGCTCACATGGAAGGTTGCGTCGGGG |  |
| **R0808_R_fwd** | CCCCGACGCAACCTTCCATGTGAGCTGAGGGAGCACCAGC |  |
| **R0808_R_rev** | GTCGACTCTAGAGGATCCCCCGGTCCTTCACTTCCCAGCG |  |
| **R3997_fwd_EcoRI** | GGGGAATTCTCACAGAGCCCAGCGCCATG | Construction of plasmid pSEVA2313_R3997 |
| **R3997_rev_HindIII** | GGGAAGCTTTCAGGCGAAGTCGAAGGTGA |  |
| **R0808_fwd_EcoRI** | GGGGAATTCGCCATCACCGAACCCCG | Construction of plasmid pSEVA2313_R0808 |
| **R0808_rev_HindIII** | GGGAAGCTTTCAGCTCACGCGGTAGCGGAAC |  |
